# MKK4/5-MPK3/6 Cascade Regulates *Agrobacterium*-Mediated Transformation by Modulating Plant Immunity in *Arabidopsis*

**DOI:** 10.3389/fpls.2021.731690

**Published:** 2021-09-30

**Authors:** Tengfei Liu, Li Cao, Yuanyuan Cheng, Jing Ji, Yongshu Wei, Chenchen Wang, Kaixuan Duan

**Affiliations:** Department of Plant Pathology, Nanjing Agricultural University, Nanjing, China

**Keywords:** *Agrobacterium*-mediated transformation, MKK4/MKK5, MPK3/MPK6, plant immunity, *Arabidopsis*

## Abstract

*Agrobacterium tumefaciens* is a specialized plant pathogen that causes crown gall disease and is commonly used for *Agrobacterium*-mediated transformation. As a pathogen, *Agrobacterium* triggers plant immunity, which affects transformation. However, the signaling components and pathways in plant immunity to *Agrobacterium* remain elusive. We demonstrate that two *Arabidopsis* mitogen-activated protein kinase kinases (MAPKKs) MKK4/MKK5 and their downstream mitogen-activated protein kinases (MAPKs) MPK3/MPK6 play major roles in both *Agrobacterium*-triggered immunity and *Agrobacterium*-mediated transformation. Agrobacteria induce MPK3/MPK6 activity and the expression of plant defense response genes at a very early stage. This process is dependent on the MKK4/MKK5 function. The loss of the function of *MKK4* and *MKK5* or their downstream *MPK3* and *MPK6* abolishes plant immunity to agrobacteria and increases transformation frequency, whereas the activation of MKK4 and MKK5 enhances plant immunity and represses transformation. Global transcriptome analysis indicates that agrobacteria induce various plant defense pathways, including reactive oxygen species (ROS) production, ethylene (ET), and salicylic acid- (SA-) mediated defense responses, and that MKK4/MKK5 is essential for the induction of these pathways. The activation of MKK4 and MKK5 promotes ROS production and cell death during agrobacteria infection. Based on these results, we propose that the MKK4/5-MPK3/6 cascade is an essential signaling pathway regulating *Agrobacterium*-mediated transformation through the modulation of *Agrobacterium-*triggered plant immunity.

## Introduction

*Agrobacterium tumefaciens* is a plant pathogen that causes crown gall disease in a wide range of species (Smith and Townsend, 1907). This pathogen has the ability to mobilize and integrate a transfer DNA (T-DNA) segment from its tumor-inducing (Ti) plasmid to the host cell genome, leading to the formation of tumorous growths (Escobar and Dandekar, 2003). The transfer of T-DNA and several virulence effector proteins to the plant through a type IV secretion system (T4SS) is essential for transformation. Although the transient expression of a T-DNA-encoded gene can occur without the integration of T-DNA into the host cell genome, genetic transformation requires T-DNA integration (Gelvin, 2017). Taking advantage of this ability, *A. tumefaciens* has been modified for use in the plant genetic engineering method of *Agrobacterium*-mediated transformation, which is better known than its role as a disease-causing bacterium (Azpiroz-Leehan and Feldmann, 1997).

Throughout the interaction between *Agrobacterium* and hosts, *Agrobacterium* infection triggers the innate immune response of the host, which is characterized by the induction of defense gene expression (Ditt et al., 2001, 2006; Veena et al., 2003; Duan et al., 2018). This response is elicited by the perception of pathogen-associated molecular patterns (PAMPs) by specific plant receptors. A primary PAMP molecule originating from *Agrobacterium* is elongation factor Tu (EF-Tu), which is perceived by the plant receptor EFR (Zipfel et al., 2006). Plant immunity induced by EF-Tu leads to reduced transformation by *Agrobacterium* (Zipfel et al., 2006). This finding demonstrates that plant immunity to *Agrobacterium* affects the transformation process. However, the detailed mechanism and signaling pathway of plant immunity to *Agrobacterium* remain unclear.

In plants, mitogen-activated protein kinase (MAPK) cascades play essential roles in the processes of plant growth and development as well as in responses to environmental stimuli such as cold, heat, drought, and especially pathogen attack (Droillard et al., 2004; Brodersen et al., 2006; Colcombet and Hirt, 2008; Meng and Zhang, 2013; Xu and Zhang, 2015). These cascades comprised of MAPK kinase (MAPKKK), MAPKK (MAPK kinase), and MAPK proteins and link upstream receptors to downstream targets *via* a phosphorylation mechanism. *Arabidopsis* MAPKKK3/5-MKK4/5-MPK3/6 form a MAPK cascade together downstream of flagellin-sensitive 2 (FLS2) (Gomez-Gomez et al., 1999; Desikan et al., 2001; Asai et al., 2002; Nimchuk et al., 2003; Pfund et al., 2004; Sun et al., 2018). Another *Arabidopsis* MAPK cascade consisting of MEKK1, MKK1/2, and MPK4 is activated during pattern-triggered immunity (PTI) (Gao et al., 2008; Qiu et al., 2008). A recent study has shown that the *Arabidopsis* MKK4/5-MPK3/6 cascade plays a major role in plant stomatal immunity (Wang et al., 2007; Su et al., 2017). Previous research indicated that agrobacteria activate MPK3 during their infection process (Pitzschke et al., 2009). Even so, the function of MPK3 in *Agrobacterium*-mediated transformation remains unclear, and whether its involved pathway plays a role in the plant response to *Agrobacterium* or the transformation process requires further investigation.

Previous research has shown that salicylic acid (SA) and ethylene (ET) function as essential signaling molecules in plant defense to *Agrobacterium* and affect transformation (Lee et al., 2009). The exogenous application of SA to *Agrobacterium* cells inhibited the expression of vir genes, affecting bacterial growth, bacterial attachment to plant cells, and virulence (Yuan et al., 2007; Anand et al., 2008). At the beginning of *Agrobacterium* infection (3-h postinfection), the level of 1-amino-cyclopropane-1-carboxylic acid (ACC), an ET precursor, is elevated in the presence of both virulent and disarmed *Agrobacterium* strains (Lee et al., 2009). The inoculation of melon explants with *Agrobacterium* also increases ET production (Ezura et al., 2000). The application of ACC reduces *Agrobacterium*-mediated transformation of melon, whereas the addition of aminoethoxyvinylglycine, an inhibitor of ACC synthase, increases transformation (Ezura et al., 2000). Thus, ET and SA derived from the host plant have effects on the defense response to *Agrobacterium* and transformation.

Here, we report that *Arabidopsis* MKK4 and MKK5, as well as their downstream kinases MPK3 and MPK6, play major roles in *Agrobacterium*-triggered immunity and transformation. They are involved in plant immunity to *Agrobacterium* through the regulation of plant defense pathways, including defense-responsive gene expression, reactive oxygen species (ROS) production, and the synthesis of ET and SA. Together, these data indicate that the MKK4/5-MPK3/6 cascade is an essential signaling pathway contributing to plant immunity to *Agrobacterium* and regulating *Agrobacterium*-mediated transformation through the modulation of *Agrobacterium*-triggered immunity in *Arabidopsis*. Based on these findings, we propose that the MKK4/5-MPK3/6 module can be modified in the future to promote the application of *Agrobacterium*-mediated transformation technology in other species.

## Materials and Methods

### Plant Materials and Growth Conditions

The *Arabidopsis thaliana* Col-0 ecotype was used in this study. *Arabidopsis* plants were grown in a growth chamber at 22°C, with 10 h-day/14 h-night photoperiod and 100 μmol m^−2^ s^−1^ light intensity. Mutants *mpk3-1* (Salk_151594), *mpk6-2* (Salk_073907), and *acs2/6/4/5/9* (CS16644) were obtained from the *Arabidopsis* Biological Resource Center (ABRC). *MPK6SR* (*mpk3 mpk6 P*_*MPK*6_*:MPK6*^*YG*^), *MKK4-DD, NahG*, and *mkk4/mkk5* double mutants were kindly provided by Dr. Suqun Zhang (University of Missouri, MO, USA). To generate *MKK5-DD* and *MEK2-DD* transgenic lines, *Arabidopsis MKK5* and tobacco *NtMEK2* complementary DNAs (cDNAs) were cloned as described in previous studies (Yang et al., 2001; Ren et al., 2002). The mutant cDNAs with a FLAG epitope at their N-termini were subcloned into the steroid-inducible pTA7002 binary vector (Aoyama and Chua, 1997). Then, the constructs were transferred into GV3101 to be transformed into Col-0 plants using the floral-dip method.

### Infection by Agrobacteria for Transcriptome and Gene Expression Analysis

For gene expression analysis, *Arabidopsis* was cultured in half-strength Murashige and Skoog (1/2 MS) liquid medium for 10 days. The resulting 10-day-old seedlings were mock treated (1/2 MS liquid medium only) or treated with 2 × 10^8^ cfu/ml GV3101 agrobacteria cells suspended in 1/2 MS liquid medium without shaking. Samples were harvested at four time points (3, 6, 12, and 24 h postinoculation (hpi) with agrobacteria). Three replicates were performed for each treatment. The seedlings were harvested at each time point and immediately frozen in liquid nitrogen; within each replicates, all seedlings were pooled together for RNA extraction and quantitative real-time PCR (qRT-PCR) analysis.

### Plant Total RNA Extraction and qRT-PCR

Total RNA was extracted from infected *Arabidopsis* seedlings using the Trizol reagent (Invitrogen, Carlsbad, CA, USA). First-strand cDNA was synthesized from the extracted messenger RNA (mRNA) using oligo dT primer and reverse transcriptase (Promega, Madison, WI, USA). qRT-PCR was performed using a Bio-Rad CFX96 Real-Time System and iQ™ SYBR® Green Supermix (Bio-Rad, Hercules, CA, USA) with 40 cycles. Templates were normalized with *Arabidopsis* UBQ10. Three biological and technical replicates were performed. Data were analyzed using the BioRad CFX Manager 2.0 Software. The comparative cycle threshold method (ΔΔCt) was used to obtain the relative fold change. Primer information is provided in Supplementary Table 1.

### RNA-Sequencing Analysis

For RNA-sequence (RNA-seq) analysis, 10-day-old *Arabidopsis* seedlings of Col-0 and *mkk4/5* were mock treated or treated with agrobacteria for 24 h. The seedlings were collected individually and frozen immediately in liquid nitrogen for RNA-seq. Three biological replicates were performed. Total RNA was used to make RNA-seq libraries with the Illumina TruSeq RNA Sample Preparation Kit v2 (Illumina, San Diego, CA, USA) following the recommendations of the manufacturer. These libraries were sequenced by Beijing Genomics Institute (BGI, Shenzhen, China).

The clean reads of all RNA-seq data obtained from BGI were mapped to *A. thaliana* TAIR10 genome by hisat2 (version 2.1.0) with default parameters. However, the above mapped SAM files were sorted and indexed by SAMtools (version 1.5). The expression data were calculated using featureCounts (v1.6.1) in default setting (Li et al., 2009; Liao et al., 2014; Kim et al., 2019). Genes with at least two-fold changes of expression in agrobacteria treatment compared to the mock treatment were identified as agrobacteria-responsive genes (ARGs). ARGs that were no longer induced by agrobacteria in *mkk4/5* were identified as MKK4/MKK5-dependent ARGs.

### Infection of Whole *Arabidopsis* Seedlings to Evaluate Transformation Frequency

To evaluate transformation frequency in whole *Arabidopsis* seedlings, the AGROBEST infection assay was used (Wu et al., 2014). Ten-day-old *Arabidopsis* seedlings grown in 1/2 MS liquid medium were infected with 10^8^ cfu/ml GV3101-pBISN1. At 3-day postinfection (dpi), seedlings were harvested for histochemical β-glucuronidase (GUS) staining and activity testing.

### *Agrobacterium-*Mediated Stable Transformation Assay

The *A. tumefaciens* tumorigenic strain A208 was used for stable transformation assays. Roots from 14-day-old *Arabidopsis* plants grown in sterile square Petri dishes containing sterile Gamborg's B5 medium were cut into ~5-mm segments. Root segments were assayed as described by Tenea et al. (2009). Tumors were scored after 30-day cultivation on MS medium. Three replicates were performed for each experiment. Statistical analysis was performed using Student's *t*-test.

### Histochemical GUS Staining and Activity Assays

For histochemical GUS staining, infected seedlings were stained in 0.2 mg/L 5-bromo-4-chloro-3-indolyl glucuronide (X-Gluc) solution at 37°C overnight and then destained with 70% ethanol for observation. β-galactosidase activity was assessed with a fluorescent substrate (4-methylumbelliferyl-β-D-glucuronide (MUG assay)) to quantitatively determine GUS activity. These assays were performed according to the method of Jefferson et al. (1987). All of these were detected at 3 days after infection. Three replicates were performed.

### Plant Total Protein Extraction and Immunoblot Analysis

Seedlings cultured for 10 days in liquid 1/2 MS medium were used for protein extraction. These 10-day-old seedlings were mock treated (1/2 MS liquid medium only) or treated with agrobacteria cells suspended in 1/2 MS liquid medium. Samples were harvested at four time points (5, 15, 30, and 60 min). Total protein was extracted from the whole seedlings in an extraction buffer [50 mM Tris-HCl at pH 7.5, 150 mM NaCl, 10 mM EDTA, 1 mM Na_3_VO_4_, 10 mM NaF, 10% glycerol, and 1 × protease inhibitor cocktail (Sigma, St. Louis, MO, USA)]. The concentration of each protein extract was determined using the Bio-Rad protein assay kit (Bio-Rad, Hercules, CA, USA) with bovine serum albumin as the standard.

For the immunoblot analysis, anti-FLAG (F3165, Sigma, St. Louis, MO, USA) and anti-pTEpY (#4696, Cell Signaling Technology, Danvers, MA, USA) antibodies were used to detect MKK4-DD, MKK5-DD, and NtMEK2-DD protein expression and MPK3/MPK6 activation, respectively.

### Leaf Infiltration for H_2_O_2_ Detection and Cell Death Analysis

The leaf infiltration of *Arabidopsis* with *Agrobacterium* GV3101 carrying pBISN1 (GV3101-pBISN1) *Arabidopsis* was performed as described previously (Wroblewski et al., 2005). Agrobacteria cells resuspended in an infiltration buffer (10 mM MES, 10 mM MgCl_2_, and 100 μM acetosyringone at pH 5.6) were injected into 4-week-old plant leaves. Then, the leaves were used for H_2_O_2_ detection and cell death analysis.

### H_2_O_2_ Detection

H_2_O_2_ was detected by the 3,3′-diaminobezidine (DAB) uptake method, which is an endogenous peroxidase-dependent *in situ* histochemical staining procedure using DAB (Thordal-Christensen et al., 1997). Leaves that were infiltrated with agrobacteria or mock infiltrated and treated with dexamethasone (DEX) were detached and placed in DAB solution (1 mg/ml, pH 5.5) for 2 h. The leaves were then boiled in ethanol (96%) for 10 min and then stored in 96% ethanol. H_2_O_2_ production is visualized as a reddish-brown coloration.

### Determination of Endogenous Levels of SA

Ten-day-old *Arabidopsis* seedlings were infected with agrobacteria cells suspended in 1/2 MS liquid medium (2 × 10^8^ cfu/ml) or mock infected (1/2 MS liquid medium only). Samples were harvested at 24 h after inoculation and frozen in liquid nitrogen. Approximately 100 mg of each sample was suspended in 90% (V/V) methanol at a ratio of 1:1000. Samples were vortexed, sonicated for 15 min, and centrifuged at 15,000 × g for 20 min two times. The supernatant was transferred to a tube, and the SA solution was filtered and separated on a C18 analytical column (Waters, Milford, MA, USA) using high performance liquid chromatography (HPLC) and detected using fluorescence (excitation at 305 nm, emission at 405 nm; Waters, Milford, MA, USA). The mobile phase used for HPLC was 90% methanol and 0.1% formic acid with a flow rate of 0.8 ml/min.

### Statistical Analysis

All data were analyzed using Student's *t*-test.

## Results

### *Agrobacterium* Infection Leads to Rapid MPK3/MPK6 Activation and Defense-Responsive Gene Induction in *Arabidopsis*

**Figure 1:**
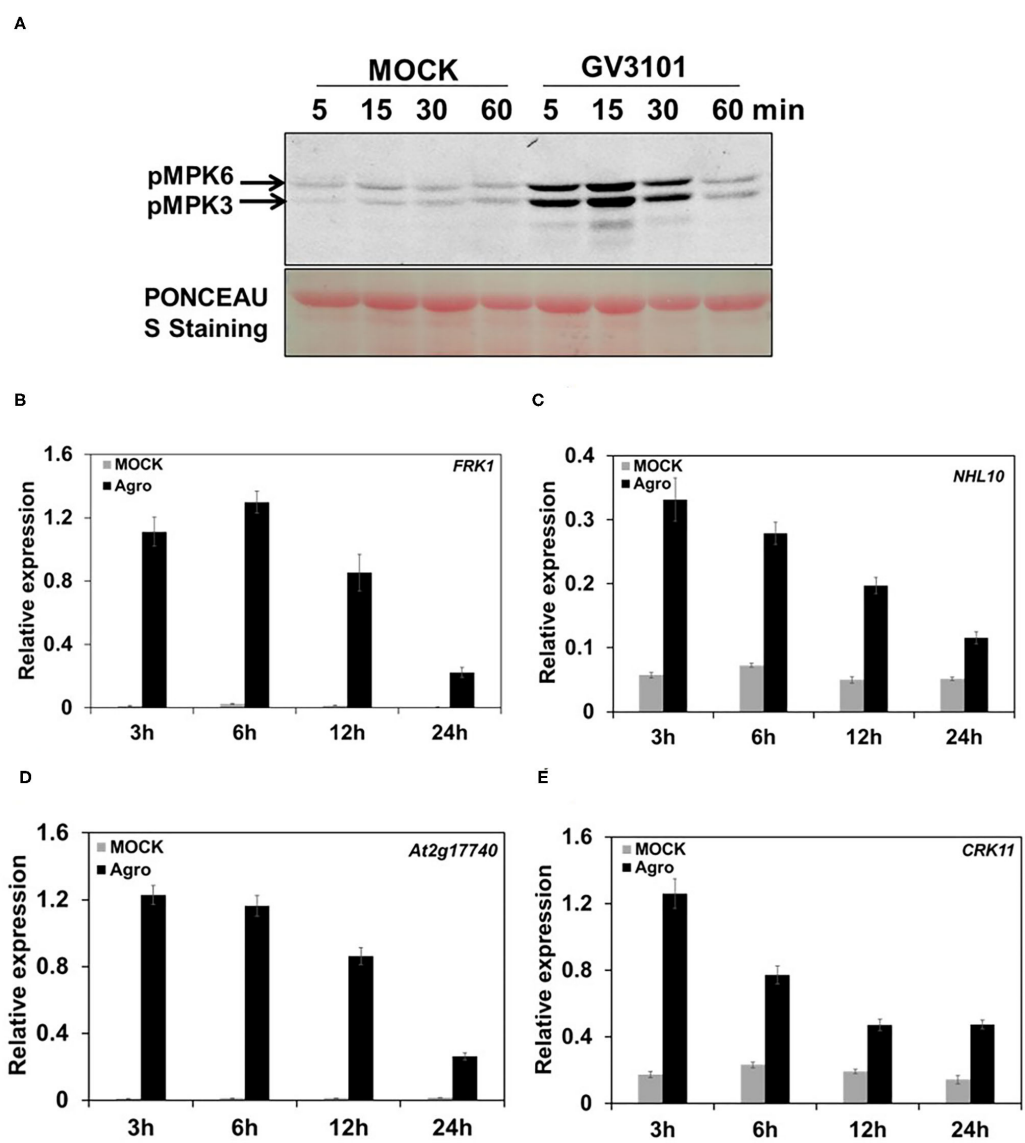
*Agrobacterium* leads to a rapid and strong MPK3/6 activation and defense-responsive gene expression. **(A)** Agrobacteria induce MPK3/MPK6 activation. Ten-day-old wild type (WT) (Col-0) seedlings grown in liquid 1/2 Murashige and Skoog (MS) medium were inoculated with mock (1/2 liquid MS medium only) and 2 × 10^8^ cfu/ml GV3101 suspension cells (suspended in 1/2 liquid MS medium) for the indicated time, respectively. Mitogen-activated protein kinase (MAPK) activation was detected by the immunoblot analysis using an anti-pTEpY antibody. Equal loading was confirmed by PONCEAU S staining. **(B–E)** Quantitative real-time PCR (qRT-PCR) analysis of some defense genes kinetic expression in WT (Col-0) infected by agrobacteria or mock treatment. Total RNA was extracted from a 10-day-old seedling treated as described in **(A)**. Relative gene expression levels are shown for: **(B)** *FRK1*, **(C)** *NHL10*, **(D)** *At2g17740*, and **(E)** *CRK11*. *UBQ10* was used as an internal control. “Agro” is an abbreviation of *Agrobacterium*. Values represent the average of three replicates with error bars indicating SD of the mean.

The activation of mitogen-activated protein kinases (MAPKs) and the induction of defense-responsive gene expression are the early steps in the immune response after plants sense an invading pathogen (Hammond-Kosack and Jones, 1996; Zhang and Klessig, 2001). To elucidate the plant immune response to *Agrobacterium*, currently we used *Arabidopsis* model to profile MPK3/MPK6 activation and defense-responsive gene expression during the *Agrobacterium* infection process. In this system, 10-day-old *Arabidopsis* seedlings of wild-type (WT) (Col-0) cultured in liquid medium were infected with the disarmed hyper-virulent *Agrobacterium* strain GV3101. Samples were harvested at various postinfection time points. Then, MPK3/MPK6 activity and defense-responsive gene expression were assessed. The results showed that, compared with mock treatment, agrobacteria infection triggered a strong and rapid activation of MPK3/MPK6 (Figure 1A). During the agrobacteria infection process, the MPK3 and MPK6 activity reached peak levels at as early as 15 min postinfection and then decreased dynamically over time (Figure 1A). Meanwhile, after mock treatment, the MPK3 and MPK6 activity remained at a stable and low level during the whole stage, which was much lower than the level after inoculation with agrobacteria and showed no changes during the whole process (Figure 1A). Interestingly, we also observed other bands besides MPK3/MPK6 after agrobacteria infection. We inferred that another MAPK MPK4 is potentially activated by agrobacteria (Figure 1A). This finding demonstrates that MPK3/MPK6 signaling is involved in the process through which plants sense agrobacteria invasion. The expression of defense-responsive genes, such as *FRK1, NHL10, At2g17740*, and *CRK11*, was measured during the agrobacteria infection process. The results showed that the expression of these genes was strongly induced by agrobacteria inoculation, especially at the early stages (e.g., 3 and 6 h postinfection), but as infection time progressed, this induction faded somewhat (Figures 1B–E). In the mock treatment, the expression of these genes remained at very low levels, with no changes during the treatment process (Figures 1B–E). Together, these results demonstrate that MPK3/MPK6 activity and defense-responsive gene expression resulted from agrobacteria infection. Thus, we conclude that *Agrobacterium* can trigger plant immune responses, characterized by a rapid activation of MPK3/MPK6 and the induction of defense-responsive gene expression.

### *Arabidopsis mkk4* and *mkk5* Are Essential to *Agrobacterium*-Triggered Immunity

**Figure 2:**
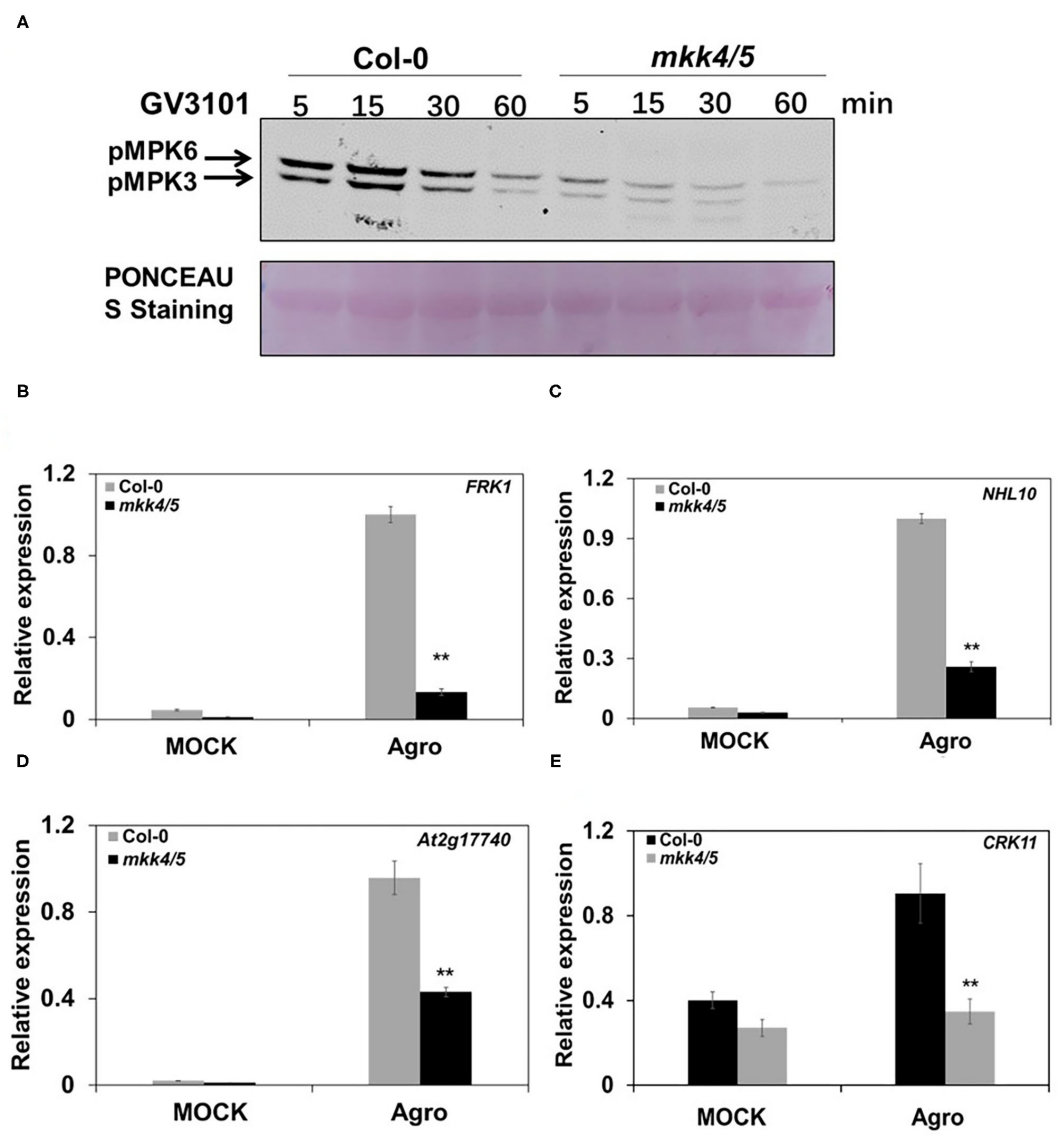
*Agrobacterium*-triggered immunity in *Arabidopsis* is dependent on MKK4 and MKK5. **(A)** The loss of the function of MKK4 and MKK5 compromises *Agrobacterium*-induced MPK3/MPK6 activation. Ten-day-old WT (Col-0) and *mkk4/5* double-mutant seedlings grown in liquid 1/2 MS medium were treated as described in Figure 1A. MAPK activation was detected by the immunoblot analysis using an anti-pTEpY antibody. Equal loading was confirmed by PONCEAU S staining. **(B–E)** The loss of the function of MKK4 and MKK5 compromises *Agrobacterium*-induced defense gene expression. Total RNA was extracted from 10-day-old WT (Col-0) and *mkk4/5* double-mutant seedlings treated as described in Figure 1A. Relative gene expression levels are shown for: **(B)** *FRK1*, **(C)** *NHL10*, **(D)** *At2g17740*, and **(E)** *CRK11*. *UBQ10* was used as an internal control. “Agro” is an abbreviation of *Agrobacterium*. Values represent the average of three replicates with error bars indicating SD of the mean. “**” indicates a significant difference at *p* < 0.01 with Student's *t*-test.

Previous studies have demonstrated that MKK4 and MKK5 are the upstream MAPKKs of MPK3/MPK6 that regulate the plant defense response and root development (Asai et al., 2002; Su et al., 2017; Shao et al., 2020). Because agrobacteria infection leads to a rapid and strong MPK3/MPK6 activation, we investigated whether MKK4 and MKK5 are required for the plant immune response to *Agrobacterium*. Here, we used *mkk4/mkk5* double mutants generated from previously reported *mkk4* and *mkk5* single mutants (Zhao et al., 2014; Su et al., 2017) to determine the functions of these two MAPKKs in *Agrobacterium*-triggered immunity. After the inoculation of 10-day-old WT (Col-0) and *mkk4/5* with the agrobacteria strain GV3101, samples were collected at various time points. MPK3/MPK6 activity and defense-responsive gene expression were measured. The results indicated that MPK3/MPK6 activity was induced and reached a high level at 15 min after the inoculation with agrobacteria in WT Col-0. However, this activity was abolished in *mkk4/mkk5* double mutants (Figure 2A). Compared with the WT, MPK3/MPK6 activity induced by agrobacteria was impaired in *mkk4/mkk5* double mutants (Figure 2A). Although some MPK3/MPK6 activities remained in *mkk4/5* double mutants, it was much weaker than in WT (Figure 2A). These results demonstrate that MKK4/MKK5 are required for the induction of MPK3/MPK6 activity by agrobacteria and act upstream of MPK3/MPK6.

Meanwhile, our results revealed that the expression of some defense-responsive genes, such as *FRK1, NHL10, At2g17740*, and *CRK11*, was significantly impaired in *mkk4/5* double mutants during agrobacteria infection, whereas the expression of those genes was strongly induced in WT, as indicated above (Figures 2B–E). Together, these results indicate that MPK3/MPK6 activity and defense-responsive gene expression induced by agrobacteria are highly dependent on the MKK4 and MKK5 function, and MKK4 and MKK5 are essential to *Agrobacterium*-triggered immunity.

### MKK4 and MKK5 Regulate *Agrobacterium-*Mediated Transformation

**Figure 3:**
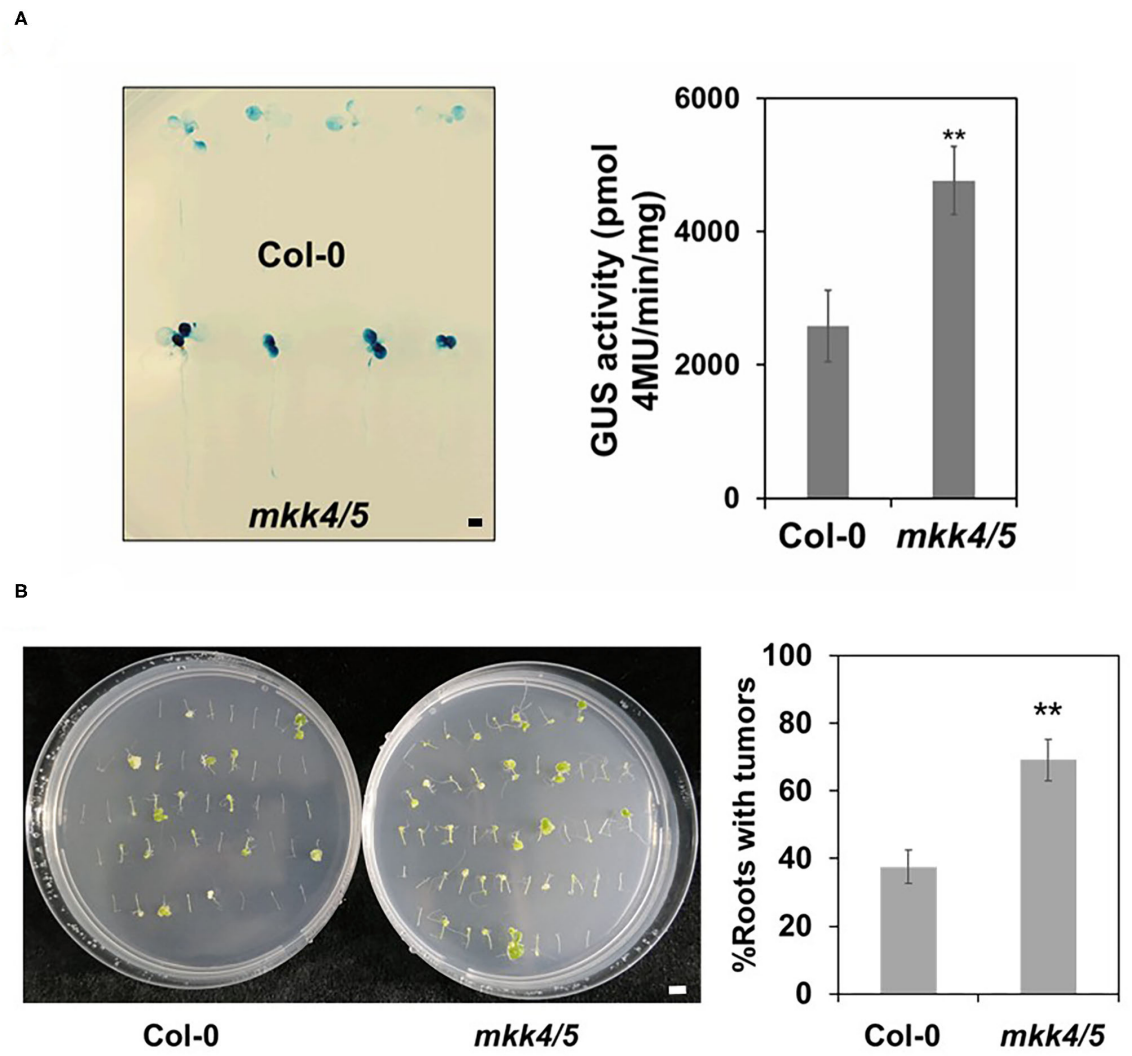
MKK4/MKK5 regulate *Agrobacterium*-mediated transformation. **(A)** *Agrobacterium*-mediated transient transformation in *mkk4/5* double mutant by using the AGROBEST system. Ten-day-old *Arabidopsis* whole seedlings of WT (Col-0) (upper panel) and *mkk4/5* double mutants (lower panel) were infected with 10^8^ cfu/ml GV3101-pBISN1 suspension cells, and transformation frequency was measured by histochemical β-glucuronidase (GUS) staining (left) and 4-methylumbelliferyl-β-D-glucuronide (MUG) assay (right) at 3-day postinfection (dpi). **(B)** *Agrobacterium*-mediated stable transformation in *mkk4/5* double mutants by using the tumorigenesis system. Root segments from 14-day-old *Arabidopsis* WT (Col-0), *mkk4/5* double-mutant plants were inoculated with 10^6^ cfu/ml of the *Agrobacterium tumefaciens* strains A208. Tumors were scored at 30 days after infection. Values represent the average of three replicates with error bars indicating SD of the mean. “**” indicates a significant difference at *p* < 0.01 with Student's *t*-test. The scale bar indicates 5 mm.

Impaired plant immunity has been found to lead to increased transformation by *Agrobacterium* (Zipfel et al., 2006). As we have established that plant immune responses to agrobacteria are impaired in the *mkk4/5* double mutants, we hypothesized that the *mkk4/5* double mutant will exhibit a phenotype of enhanced *Agrobacterium*-mediated transformation. Hence, we employed two different transformation systems, namely transient (AGROBEST) and stable transformation (tumorigenesis induced by agrobacteria), to evaluate how the impaired immunity of this mutant affects the transformation. Firstly, we evaluated the transient transformation frequency in the whole seedling using the AGROBEST infection method (Wu et al., 2014). Ten-day-old seedlings of WT (Col-0) and *mkk4/5* double mutants cultured in liquid medium were infected with the hyper-virulent nontumorigenic *Agrobacterium* strain GV3101 containing the binary vector pBISN1-GUS, which carries a GUS intron gene allowing its expression only in plants but not in *Agrobacterium* (GV3101-pBISN1). Then, GUS activity was assessed to monitor the transient expression efficiency at 3 dpi. Compared with WT plants, stronger and more concentrated spots of GUS staining were detected in *mkk4/5* double-mutant seedlings at 3 dpi (Figure 3A). The enzymatic activity of GUS was ~1.8-fold greater in *mkk4/5* double mutants than in WT (Figure 3A). Moreover, stable transformation *via* tumorigenesis was tested to confirm the function of MKK4/MKK5 in transformation. Root segments were infected with the tumorigenic *A. tumefaciens* strain A208 (Nam et al., 1997; Shi et al., 2014), and the root tumorigenesis efficiency was analyzed. Our results showed that compared to the WT, the *mkk4/5* mutant exhibited ~1.8-fold greater tumorigenesis efficiency (Figure 3B), supporting the hypothesis that MKK4/MKK5 signaling is an important pathway in *Agrobacterium*-mediated transformation *via* the regulation of plant immunity.

### Gain-of-Function Activation of MKK4 or MKK5 Increases Plant Immunity to *Agrobacterium* and Represses Transformation

**Figure 4:**
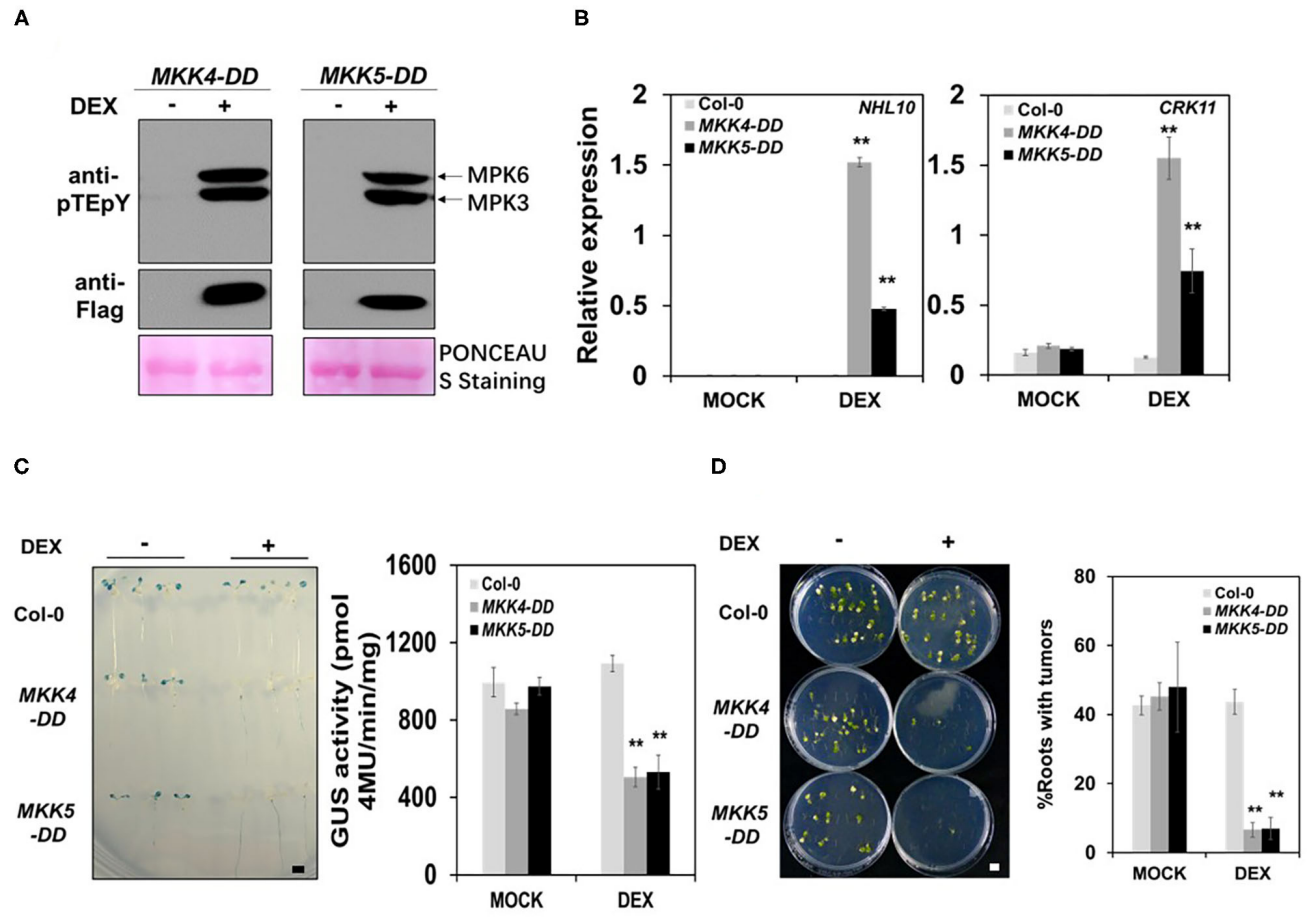
The activation of MKK4 or MKK5 in *Arabidopsis* increases the plant immunity to *Agrobacterium* and represses the transformation. **(A)** MKK4-DD or MKK5-DD protein expression and MPK3/MPK6 activation in *MKK4-DD* and *MKK5-DD* transgenic seedlings after induction by dexamethasone (DEX). Ten-day-old seedlings grown in 1/2 MS liquid medium were treated with mock (ethanol) or DEX (5 μM) for overnight before test. The induction of MKK4-DD or MKK5-DD expression and MPK3/MPK6 activation were detected by the immunoblot analysis using anti-FLAG and anti-pTEpY antibodies, respectively. Equal loading was confirmed by PONCEAU S staining. **(B)** The induction of defense gene expressions in *MKK4-DD* and *MKK5-DD* transgenic seedlings. After DEX pretreatment, total RNA was extracted from 10-day-old WT (Col-0), *MKK4-DD*, and *MKK5-DD* transgenic plants. Transcript levels were determined by qRT-PCR for NHL10 (left) and CRK11 (right). *UBQ10* was used as an internal control. **(C)** The evaluation of transformation frequency in *MKK4-DD* or *MKK5-DD* transgenic seedlings compared with WT (Col-0) by using the AGROBEST system. After pretreatment with mock (ethanol) or DEX, 10-day-old *Arabidopsis* whole seedlings of WT (Col-0), *MKK4-DD*, and *MKK5-DD* transgenic lines were infected with 10^8^ cfu/ml GV3101-pBISN1 suspension cells, and transformation frequency was measured by histochemical GUS staining (left) and MUG assay (right) at 3 dpi. **(D)** Stable transformation frequency of *MKK4-DD* or *MKK5-DD* transgenic seedlings compared with WT (Col-0) by using the tumorigenesis system. After pretreatment with mock (ethanol) or DEX, root segments from 14-day-old *Arabidopsis* WT (Col-0), *MKK4-DD* and *MKK5-DD* transgenic plants were inoculated with 10^6^ cfu/ml of the *A. tumefaciens* strains A208. Tumors were scored at 30 days after infection. Values represent the average of three replicates with error bars indicating SD of the mean. “**” indicates a significant difference at *p* < 0.01 with Student's *t*-test. The scale bar indicates 5 mm.

Previous studies have demonstrated that gain-of-function activation of *MKK4* or *MKK5* were effectively inducing the plant defense responses in tobacco and *Arabidopsis* using transgenic lines with DEX-inducible MKK4 and MKK5 activities, respectively (Asai et al., 2002; Ren et al., 2002). To further elucidate the functions of MKK4 and MKK5 in the plant immune response to *Agrobacterium* and transformation, we analyzed the transgenic lines with DEX-inducible activity of both *MKK4* and *MKK5*, designated as *MKK4-DD* and *MKK5-DD*, in which *MKK4/MKK5* gene expression was induced by DEX. Firstly, we tested MPK3/MPK6 activation in DEX-inducible *MKK4-DD* and *MKK5-DD* transgenic lines. The results showed a strong MPK3/MPK6 activation in *MKK4-DD* and *MKK5-DD* transgenic lines after DEX treatment (Figure 4A), indicating that active MKK4/MKK5 can lead to the activation of MPK3/MPK6 and confirming that MKK4/MKK5 function upstream of MPK3/MPK6. The expression of defense-responsive genes, such as *CRK11* and *NHL10*, was also tested in the *MKK4-DD* and *MKK5-DD* transgenic lines. After DEX treatment, *CRK11* and *NHL10* expression was dramatically induced in the *MKK4-DD* and *MKK5-DD* transgenic lines compared with in the WT, whereas DEX itself had no direct effect on the expression of these two genes in the WT (Figure 4B), indicating that DEX-induced defense-responsive gene expression in *MKK4-DD* and *MKK5-DD* plants resulted from MKK4/MKK5 activation.

Meanwhile, we assessed the *Agrobacterium*-mediated transformation frequency of the *MKK4-DD* and *MKK5-DD* transgenic lines. Using the AGROBEST method, WT (Col-0), *MKK4-DD*, and *MKK5-DD* transgenic plants were infected with GV3101-pBISN1. The GUS staining results showed that in DEX-treated samples, *MKK4-DD* and *MKK5-DD* plants exhibited a much lower transformation frequency at 3 dpi (Figure 4C). The measurement of GUS enzymatic activity showed that *MKK4-DD* and *MKK5-DD* plants had ~two-fold lower levels than WT after DEX treatment (Figure 4C). DEX had no effect on transformation frequency in WT (Figure 4C), demonstrating that the DEX-induced reduction of transformation frequency in *MKK4-DD* and *MKK5-DD* plants resulted from increased MKK4/MKK5 activation. Additionally, the tumorigenesis analysis was used to evaluate the stable transformation frequency of *MKK4-DD* and *MKK5-DD* transgenic plants. The tumorigenesis analysis showed the lower transformation frequency in *MKK4-DD* and *MKK5-DD* plants after DEX treatment, with fewer tumors in roots compared with WT (Figure 4D). Together, these results indicate that the activation of MKK4/MKK5 represses the transformation by agrobacteria, which may be due to the increases in MPK3/MPK6 activity and plant immunity.

Tobacco *NtMEK2* is a homolog of *Arabidopsis MKK4* and *MKK5* (Ren et al., 2002). Previous research demonstrated that after DEX treatment, gain-of-function *GVG-NtMEK2*^*DD*^ transgenic *Arabidopsis* had high activation levels of MPK3 and MPK6 (Su et al., 2017), showing similar results to the MPK3/MPK6 activation pattern in *MKK4-DD* and *MKK5-DD* plants. As a result, we inferred that *NtMEK2-DD* transgenic *Arabidopsis* showed a similar response to agrobacteria. Unsurprisingly, when treated with DEX, *NtMEK2-DD* transgenic plants exhibited strong MPK3 and MPK6 activities (Supplementary Figure 1). After DEX treatment, *NtMEK2-DD* transgenic plants had much higher expression levels of *CRK11* and *NHL10* compared with WT, and DEX alone had no effect on WT (Supplementary Figure 1).

Similarly, transformation frequency was evaluated in *NtMEK2-DD* transgenic seedlings. In 3 days after inoculation with GV3101-pBISN1, the seedlings were stained in X-Gluc solution overnight. GUS staining appeared in more areas in WT (Col-0) seedlings than in *NtMEK2-DD* transgenic seedlings after DEX treatment (Supplementary Figure 1). The detection of GUS activity indicated 2.3-fold lower activity in *NtMEK2-DD* transgenic seedlings compared with the WT (Col-0) after DEX induction (Supplementary Figure 1). GUS staining and activity showed no changes in WT (Col-0) with DEX treatment, indicating that the lower transformation frequency in *NtMEK2-DD* transgenic seedlings resulted from DEX-induced MKK4/MKK5 activation.

Together, these results support the conclusion that MKK4/MKK5 and their homolog NtMEK2 play a positive role in plant immunity to *Agrobacterium* by activating MPK3/MPK6. The activation of MPK3/MPK6 and of downstream defense pathways are the important processes in the repression of transformation by *Agrobacterium*.

### *Arabidopsis* MPK3 and MPK6 Act Downstream of MKK4 and MKK5 in *Agrobacterium-*Mediated Transformation

**Figure 5:**
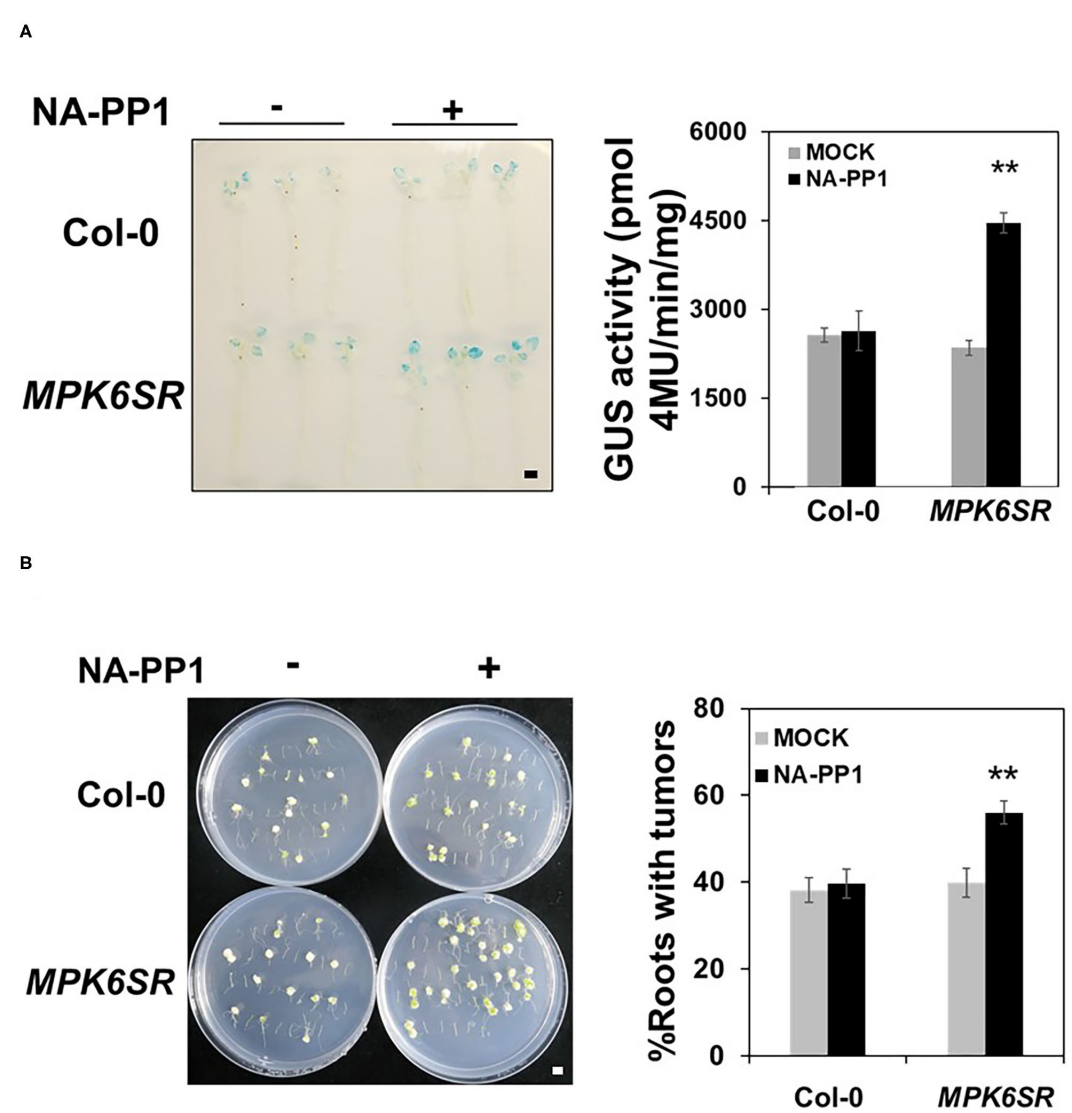
MPK3/MPK6 regulate *Agrobacterium*-mediated transformation. **(A)** The loss of the function of *MPK3* and *MPK6* increases plant transformation by *Agrobacterium* when tested with the AGROBEST system. WT (Col-0) and *MPK6SR* 10-day-old seedlings were pretreated with mock (dimethyl sulfoxide (DMSO) control) or NA-PP1 (15 μM) for overnight and then inoculated with 10^8^ cfu/ml GV3101-pBISN1 suspension cells. GUS staining (left) and MUG assay (right) were measured at 3 dpi. **(B)** The loss of the function of *MPK3* and *MPK6* increases *Agrobacterium*-mediated stable transformation by using the tumorigenesis system. WT (Col-0) and *MPK6SR* 14-day-old plants were pretreated with mock (DMSO control) or NA-PP1 (15 μM) for overnight, and root segments were inoculated with 10^6^ cfu/ml of the *A. tumefaciens* strains A208. Tumors were scored at 30 days after infection. Values represent the average of three replicates with error bars indicating SD of the mean. “**” indicates a significant difference at *p* < 0.01 with Student's *t*-test. The scale bar indicates 5 mm.

MKK4 and MKK5 are the upstream MAPKKs of MPK3/MPK6 in the regulation of plant defense responses (Asai et al., 2002; Su et al., 2017), and our data demonstrate that MKK4 and MKK5 also function upstream of MPK3/MPK6 in *Agrobacterium*-triggered immunity. Based on these results, we investigated whether these two MAPKs MPK3/MPK6 also regulate plant transformation by *Agrobacterium*. Our results reveal no significant transformation difference in *mpk3* and *mpk6* single mutants compared with the WT after inoculation with *Agrobacterium* GV3103-pBISN1 (Supplementary Figure 2), indicating that functional redundancy between MPK3 and MPK6 may exist in *Agrobacterium*-mediated transformation. As a result, a conditional *mpk3/mpk6* double-mutant, *MPK6SR*, which is sensitive to NA-PP1 (a kinase inhibitor) (Su et al., 2017; Shao et al., 2020), was used for the analysis of *Agrobacterium*-mediated transformation in *Arabidopsis*. NA-PP1 makes the activity of *MPK6SR* plants nulls that are equivalent to *mpk3*/*mpk6* double mutants (Xu et al., 2014; Su et al., 2017; Shao et al., 2020). Using this genetic material, we tested the frequency of transformation by *Agrobacterium*. After inoculation with GV3101-pBISN1, GUS staining and activity were detected in WT and *MPK6SR* seedlings. Compared with the WT, *MPK6SR* seedlings exhibited a higher transformation frequency, with a stronger GUS staining, and a 1.7-fold increase in GUS activity after treatment with NA-PP1 (Figure 5A). In the absence of the inhibitor NA-PP1, *MPK6SR* seedlings behaved like WT control (Figure 5A). Moreover, NA-PP1 itself had no effect on the transformation in WT (Col-0), demonstrating the specificity of NA-PP1 in plant transformation to *MPK6SR* lines.

We confirmed this result through the tumorigenesis analysis. As shown in Figure 5B, compared with WT, NA-PP1-treated *MPK6SR* roots had a much higher stable transformation frequency (Col-0), with more tumors in the roots. NA-PP1 itself had no effect on tumorigenesis in WT (Col-0). These results indicate that MPK3 and MPK6 act downstream of MKK4 and MKK5 to regulate *Agrobacterium*-mediated transformation.

### Global Transcriptome Analysis Indicated MKK4/MKK5 Regulate Plant Defense Pathways During *Agrobacterium-*Mediated Transformation

**Figure 6:**
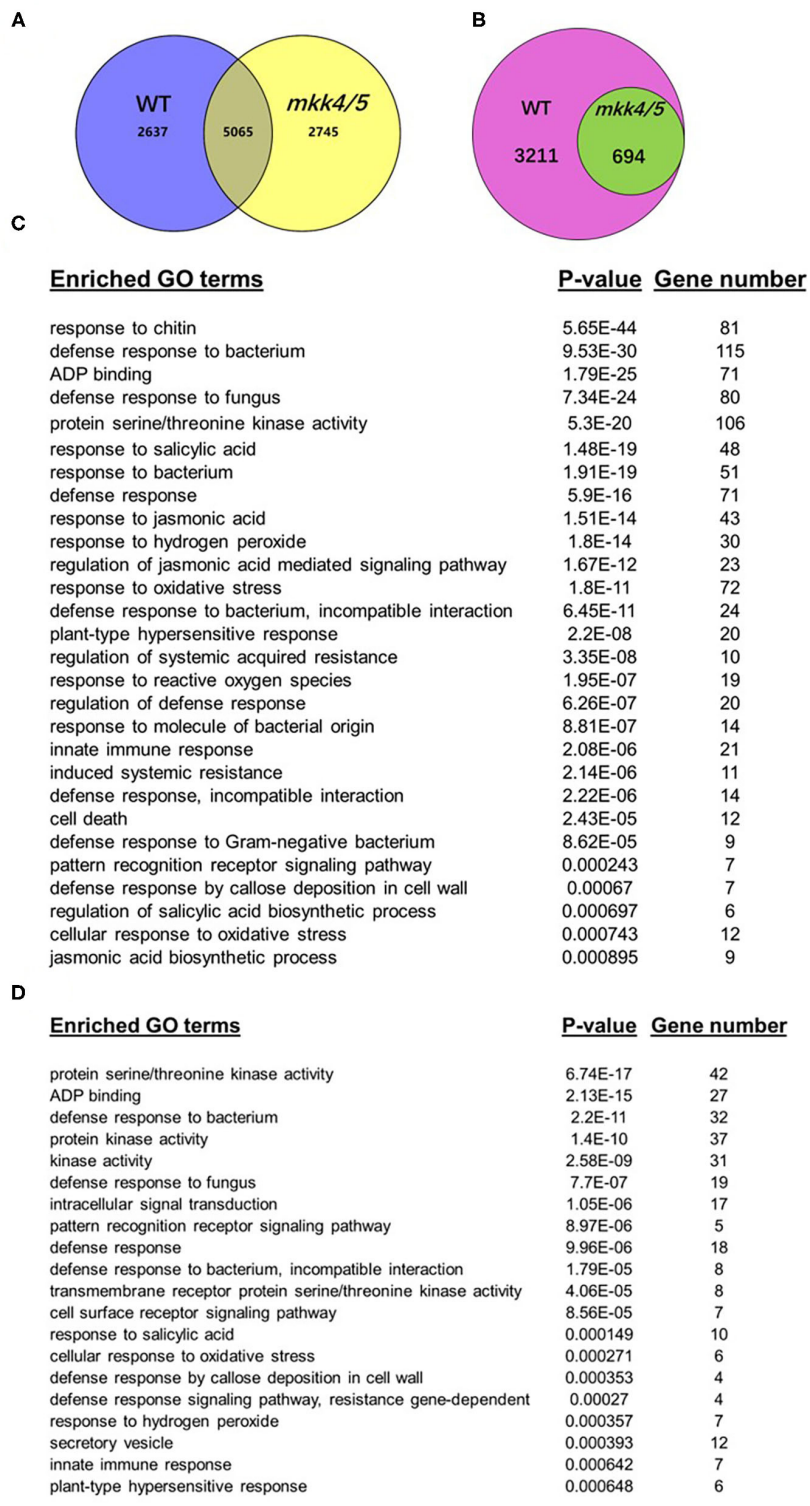
Global transcriptome analysis indicates that *Agrobacterium-*triggered plant defense pathways are dependent on MKK4/MKK5 function. **(A)** A Venn diagram displaying sets of differentially expressed genes in the WT (blue circle) or *mkk4/5* double mutant (yellow circle) to the comparisons between the treatment with mock and *Agrobacterium* strain GV3101. **(B)** A Venn diagram displaying sets of upregulated genes after agrobacteria treatment in WT (pink circle) while no expression changes in *mkk4/5* double mutants (green circle). **(C)** Selected overrepresented gene ontology (GO) biological process categories in WT induced by agrobacteria treatment. Sets of genes used to determine significantly enriched GO terms were those found to be differentially expressed at *p* < 0.05 significance level and showed at least a two-fold change in expression. The included categories are those found to be significantly enriched (*p* < 0.01) among upregulated gene in agrobacteria treatment. **(D)** Selected overrepresented GO biological process categories, which are induced by agrobacteria in WT while no changes in *mkk4/5* double-mutant plants. *P*-values from each category enrichment test have been negatively log10-transformed and plotted. “Gene number” indicates how many genes involved in each categories.

Throughout the *Agrobacterium*-mediated transformation process, the host plants exhibit dynamic gene expression patterns in response to *Agrobacterium*. Changes in host gene expression patterns throughout the transformation process have effects on transformation frequency. To clarify the mechanisms of MKK4/MKK5 in regulating the *Agrobacterium*-mediated transformation process, global transcriptome analysis was performed to compare WT and *mkk4/5* double-mutant plants. After data analysis, genes were considered significantly differentially expressed if they had a false discovery rate of less than 0.05 and a change in expression between the two treatments of at least two-fold. Overall, 7,702 unique genes in WT (blue circle) and 7,810 (yellow circle) in *mkk4/5* were differentially expressed in the agrobacteria treatment relative to the mock treatment (Figure 6A). Of these genes, 5,065 were differentially expressed between treatments in both WT and *mkk4/5* (Figure 6A). In total, 3,905 unique genes were induced by agrobacteria treatment relative to the mock treatment in WT, of which 694 showed no expression changes in *mkk4/5* plants between the agrobacterium and mock treatments (Figure 6B). This finding indicates that these 694 genes are regulated by MKK4/MKK5 in the agrobacteria infection process and may be the major factors involved in the regulation of transformation by MKK4/MKK5.

We employed the gene ontology (GO) analysis to categorize the 3,905 agrobacteria-induced genes in WT to investigate wide-scale changes in gene expression related to specific cellular and biological processes. Figure 6C shows the degree to which selected GO biological process categories were overrepresented. Categories including “defense response to bacteria,” “response to SA,” “response to hydrogen peroxide,” “cellular response to oxidative stress,” “cell death,” and “response to jasmonic acid” are overrepresented among agrobacteria-induced genes in WT (Figure 6C, Supplementary Figure 3, Supplementary Material 1), indicating that agrobacteria induce a broad range of plant immune responses. In contrast, 694 of these genes, which were no expression changes in *mkk4/5* mutant plants, were also categorized. The categories “defense response to bacteria,” “response to SA,” “response to hydrogen peroxide,” and “innate immune response” are overrepresented among these genes showing no change in expression (Figure 6D, Supplementary Figure 4, Supplementary Material 2). Thus, most plant defense pathways induced by agrobacteria were abolished in *mkk4/5* mutant plants, further confirming that MKK4/MKK5 signaling is indispensable in the plant immune response to agrobacteria. Together, these results demonstrate that agrobacteria infection generally induces plant stress and defense response pathways, which are dependent on MKK4/MKK5 activity.

### MKK4/MKK5 Regulate ROS Production and Cell Death in Agrobacteria Infection

**Figure 7:**
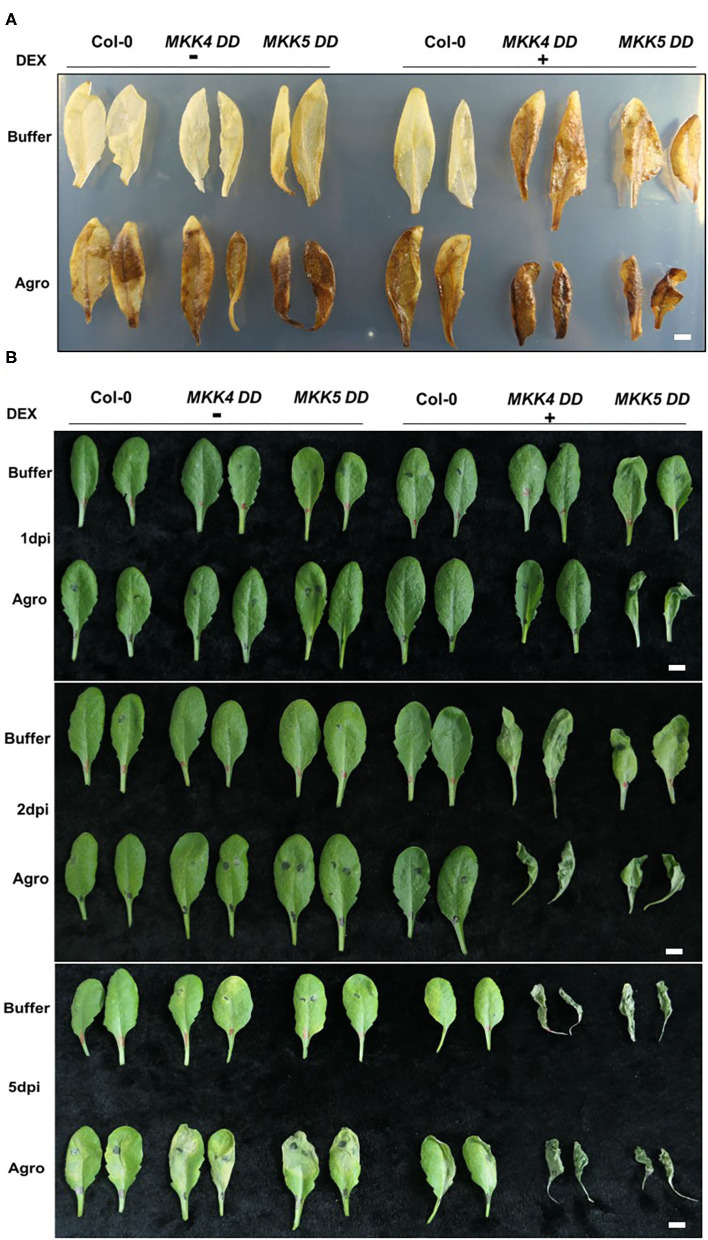
MKK4/MKK5 regulate reactive oxygen species (ROS) production and cell death during *Agrobacterium* infection process. **(A)** The activation of MKK4/MKK5 promotes the generation of H_2_O_2_ during the agrobacteria infection process. *Arabidopsis* Col-0, *MKK4-DD*, and *MKK5-DD* transgenic plants were sprayed with 15 μM DEX. After overnight of DEX treatment, the plant leaves were infiltrated with infiltration buffer (buffer) or GV3101 suspension cells (suspended in infiltration buffer). The H_2_O_2_ generation was detected by immersing the leaves in 3,3′-diaminobezidine (DAB) solution (1 mg/ml, pH 5.5) for 2 h. After the leaves were boiled in ethanol (70%) for 10 min to remove the chlorophyll, H_2_O_2_ production was visualized as a reddish-brown coloration. **(B)** The activation of MKK4/MKK5 promotes the cell death during the agrobacteria infection process. Col-0, *MKK4-DD*, and *MKK5-DD* transgenic plants were pretreated with 15 μM DEX. After DEX treatment, the plant leaves were infiltrated with infiltration buffer (buffer) or GV3101 suspension cells (suspended with infiltration buffer). Infiltrated leaves were collected at indicated times (1, 2, and 5 dpi). Cell death was observed and the photographs were taken. The scale bar indicates 5 mm.

The production of ROS and cell death are active defense mechanisms of plants against invading pathogens. Our global transcriptome data indicated that the “response to hydrogen peroxide,” “cellular response to oxidative stress,” and “cell death” pathways were induced during the agrobacteria infection process, indicating that plants produced ROS and underwent cell death to counteract agrobacteria infection. To confirm the function of MKK4/MKK5 in *Agrobacterium*-mediated ROS production and cell death, leaves from a 4-week-old seedling of WT (Col-0), *MKK4-DD*, and *MKK5-DD* were inoculated with agrobacteria GV3101 through leaf infiltration, and then the H_2_O_2_ production and cell death were assessed. As shown in Figure 7A (upper layer), in the absence of agrobacteria, the reddish-brown precipitants of oxidized DAB, an indication of H_2_O_2_ production, were visible in the leaves of *MKK4-DD, MKK5-DD* transgenic plants pretreated with DEX as an inducer. As controls, the leaves of Col-0 plants were pretreated with DEX and processed alongside inoculated leaves, and no H_2_O_2_ generation was observed in these control leaves. The leaves from Col-0, *MKK4-DD*, and *MKK5-DD* transgenic plants clearly exhibited no H_2_O_2_ generation in the absence of DEX (Figure 7A, upper layer). However, in the presence of agrobacteria, the leaves of Col-0, *MKK4-DD*, and *MKK5-DD* transgenic plants all produced H_2_O_2_ (Figure 7A, lower layer). In this analysis, H_2_O_2_ production was also detected when the plants were pretreated with DEX, and the resulting H_2_O_2_ production was much greater in *MKK4-DD* and *MKK5-DD* transgenic plants than in plants treated only with DEX (Figure 7A), indicating that agrobacteria infection can induce ROS production in plants and that DEX-induced MKK4 and MKK5 activation promotes ROS production during the agrobacteria infection process.

Similarly, cell death during the agrobacteria infection process in Col-0, *MKK4-DD*, and *MKK5-DD* transgenic plants was investigated. As shown in Figure 7B, among the leaves of Col-0, *MKK4-DD*, and *MKK5-DD* transgenic plants pretreated with DEX for 1 day but not inoculated with agrobacteria, only *MKK5-DD* plants showed very low levels of cell death while all plants without DEX treatment had no cell death. In the presence of agrobacteria inoculation at 1 dpi or 1 day after DEX treatment, the leaves of Col-0, *MKK4-DD*, and *MKK5-DD* transgenic plants without DEX treatment had no cell death; however, after pretreatment with DEX, enhanced cell death was observed in *MKK5-DD* transgenic plants, with no cell death observed in Col-0, *MKK4-DD* plants (Figure 7B). At 2 dpi or 2 days after DEX treatment, in the absence of agrobacteria inoculation, the leaves from *MKK4-DD* and *MKK5-DD* transgenic plants pretreated with DEX showed higher levels of cell death compared to Col-0 plants, and no cell death occurred in plants without DEX treatment. However, in the presence of agrobacteria inoculation, cell death was enhanced in DEX pretreated *MKK4-DD* and *MKK5-DD* transgenic plant leaves compared to leaves without agrobacteria inoculation. As controls, no cell death was observed in Col-0, *MKK4-DD*, and *MKK5-DD* transgenic plants without DEX pretreated and agrobacteria inoculation. At 5 dpi or 5 days after DEX treatment, cell death was observed in *MKK4-DD*, and *MKK5-DD* transgenic plants when pretreated with DEX and in DEX pretreated agrobacteria-infiltrated leaves. As controls, agrobacteria-infiltrated Col-0, *MKK4-DD*, and *MKK5-DD* plants also exhibited clear cell death, whereas no cell death was observed in buffer-infiltrated Col-0, *MKK4-DD*, or *MKK5-DD* plants.

Based on these results, we conclude that agrobacteria can induce plant ROS production at an early infection stage (1 dpi) and cell death at a late infection stage (5 dpi), and that the activation of MKK4/MKK5 promotes agrobacteria-induced ROS production and cell death. Thus, ROS production and cell death are involved in plant immunity and regulated by MKK4/MKK5. This pathway may be one factor influencing *Agrobacterium*-mediated transformation.

### MKK4/MKK5 Regulate SA Synthesis in *Agrobacterium-*Mediated Transformation

**Figure 8:**
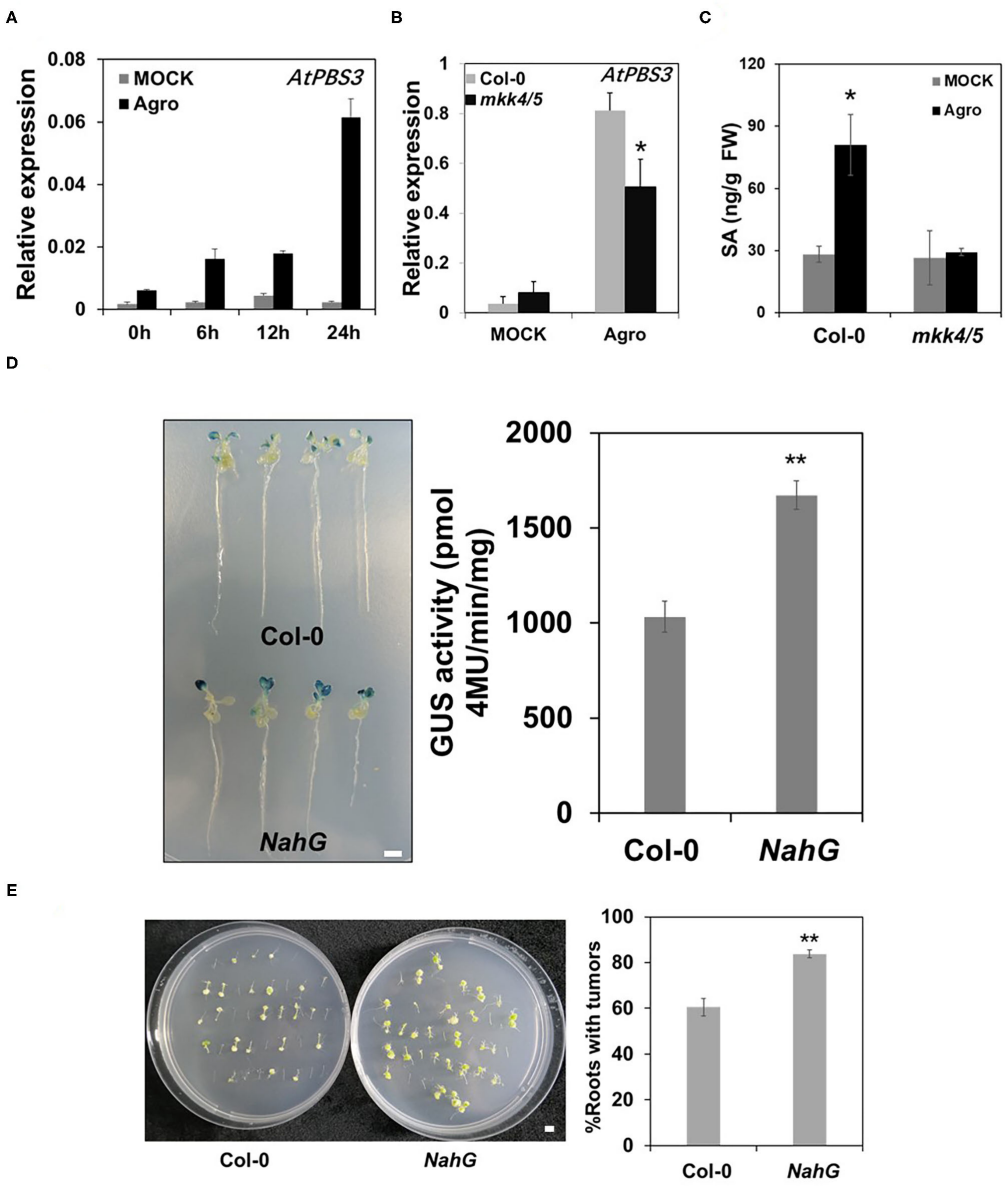
MKK4/MKK5 pathway regulates salicylic acid (SA) synthesis in *Agrobacterium*-mediated transformation. **(A)** Agrobacteria infection induces SA synthesis gene expression. Ten-day-old seedlings of WT grown in 1/2 MS liquid medium were treated with mock or 2 × 10^8^ cfu/ml GV3101 suspension cells as the indicated time point. Then, total RNA was extracted, and the transcript level was analyzed by qRT-PCR for *AtPBS3*. **(B)** The loss of the function of *MKK4* and *MKK5* compromises *AtPBS3* expression in the *Agrobacteria* inoculation. Total RNA was extracted from 10-day-old WT (Col-0) and *mkk4/5* double-mutant seedlings after treatment with agrobacteria. Relative gene expression levels are shown. *UBQ10* was used as an internal control. **(C)** SA contents in WT and *mkk4/5* after inoculation with agrobacteria. Ten-day-old WT and *mkk4/5* seedlings were treated as Figure 8A, and SA contents were measured by high performance liquid chromatography (HPLC). **(D)** *Agrobacterium*-mediated transit transformation in an SA-defective mutant (*NahG*) by using the AGROBEST system. WT (Col-0), *NahG* mutant 10-day-old seedlings were inoculated with 10^8^ cfu/ml GV3101-pBISN1 suspension cells. GUS staining (left) and MUG assay (right) were measured at 3 dpi. **(E)** *Agrobacterium*-mediated stable transformation in *NahG* by using the tumorigenesis system. Root segments from the WT (Col-0) and *NahG* mutant were inoculated with 10^6^ cfu/ml of the *A. tumefaciens* strains A208. Tumors were scored at 30 days after infection. Values represent the average of three replicates with error bars indicating SD of the mean. “**” indicates a significant difference at *p* < 0.01. The scale bar indicates 5 mm.

Salicylic acid is an important compound in plant defense (Vlot et al., 2009). Previous research demonstrated that plants with constitutively active MPK3 plants accumulate SA (Geno et al., 2017). SA accumulated at 6 days after inoculation with agrobacteria (Lee et al., 2009). Our transcriptome data indicated that SA signaling is a plant defense response to *Agrobacterium*. To explore whether SA is involved in MKK4/MKK5-regulated *Agrobacterium*-mediated transformation, we first examined the expression of SA synthesis genes *AtPBS3, AtICS1, AtEDS5* (Rekhter et al., 2019; Torrens-Spence et al., 2019) in the WT, and *mkk4/5* double mutant. As shown in Figures 8A,B, *AtPBS3* was induced after agrobacteria inoculation in the WT (Col-0), but this induction effect was impaired in *mkk4/5* double mutants. *AtICS1* and *AtEDS5* gene expressions were induced by agrobacteria inoculation in the WT, but the expression of *AtICS1* was higher in the *mkk4/5* mutant than in WT and *AtEDS5* expression showed no difference between *mkk4/5* double mutants and the WT (Col-0) mutant (Supplementary Figure 5). We then measured SA levels in plants when inoculated with agrobacteria. Whole WT (Col-0) and *mkk4/5* seedlings were harvested after inoculation with agrobacteria, and their SA contents were determined *via* HPLC. No significant difference between the WT and *mkk4/5* was found in the absence of agrobacteria inoculation (Figure 8C). Agrobacteria inoculation induced SA accumulation in WT, but this induction was abolished in the *mkk4/5* mutant (Figure 8C), indicating that the loss of the function of *MKK4/MKK5* resulted in impaired SA induction by agrobacteria in *Arabidopsis*. These results demonstrate that MKK4/MKK5 regulate the agrobacteria induction of SA synthesis during the infection process.

As SA synthesis is regulated by MKK4/MKK5 during agrobacteria infection, we next analyzed the effect of exogenous SA on transformation in WT and *mkk4/5* double mutants. The results showed that applying SA to WT (Col-0) and *mkk4/5* double-mutant seedlings caused the transformation frequency of both the WT (Col-0) and *mkk4/5* to decrease relative to plants without SA treatment, with ~1.6- and 1.8-fold decreases, respectively, in GUS activity (Supplementary Figure 5). This result demonstrates that exogenous SA rescues the phenotype of *mkk4/5* and greater SA concentration represses agrobacteria infection in *Agrobacterium*-mediated transformation. To obtain more evidence supporting this hypothesis, we examined the transformation of SA-deficient mutant *NahG* plants (van Wees and Glazebrook, 2003). The results indicated that, at 3 days after inoculation with GV3101-pBISN1, GUS staining was much stronger in *NahG* seedlings than in WT (Col-0), with a GUS activity increase of 2.9-fold compared with the WT (Col-0) (Figure 8D). Stable transformation *via* tumorigenesis was also analyzed. WT and *NahG* root segments were infected with the tumorigenic *A. tumefaciens* strain A208, and tumorigenesis efficiency was counted. The data show that compared with WT, the *NahG* mutant exhibited a much more agrobacteria-sensitive phenotype and had more tumors in roots (Figure 8E). From these results, we can conclude that SA accumulation during agrobacteria infection is regulated by MKK4/MKK5 and is involved in *Agrobacterium*-mediated transformation.

### MKK4/MKK5 Regulate ET Synthesis in *Agrobacterium-*Mediated Transformation

**Figure 9:**
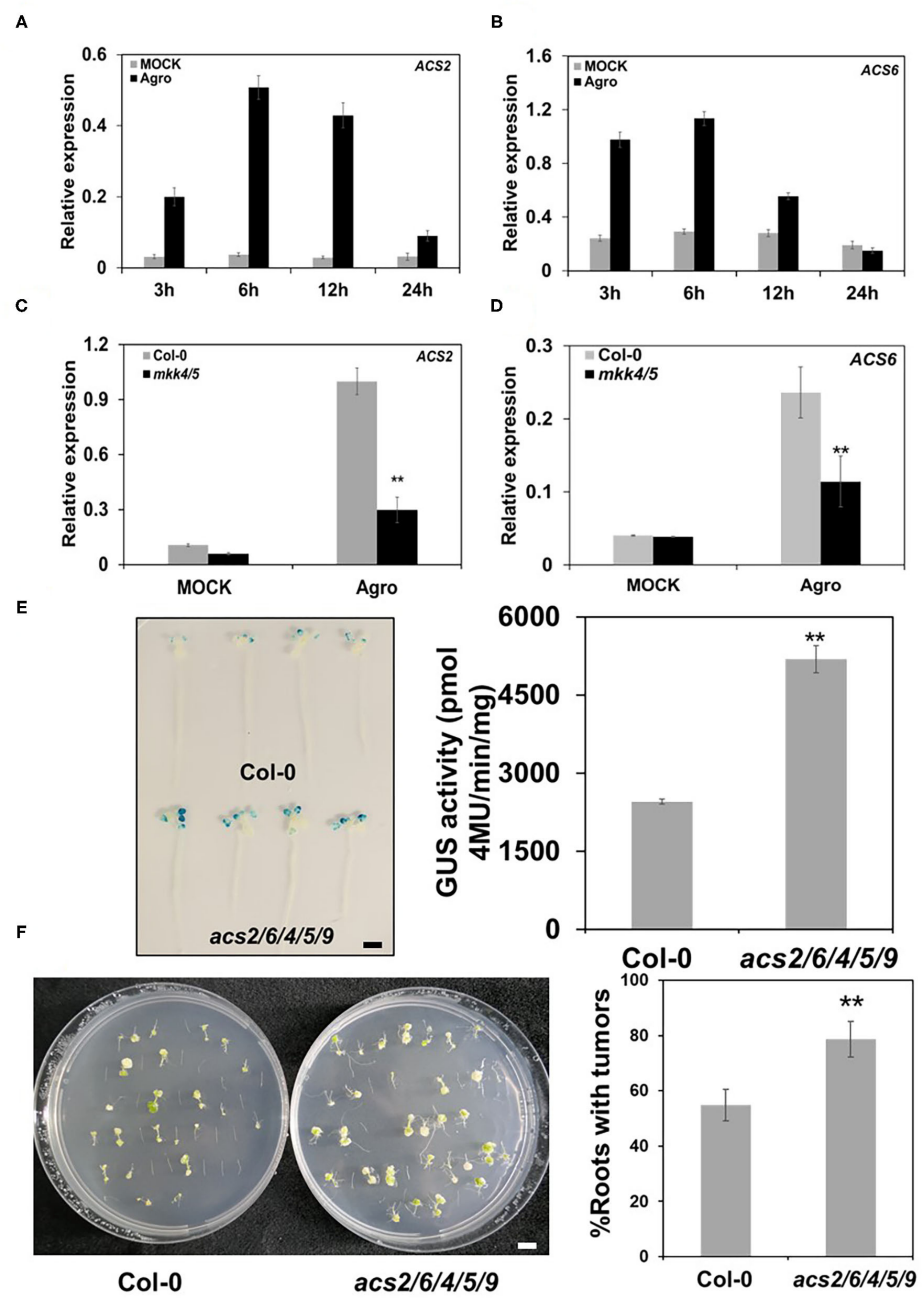
MKK4/MKK5 pathway regulates ethylene (ET) synthesis in *Agrobacterium*-mediated transformation. **(A, B)** ET synthesis genes kinetic expression in WT during the agrobacteria infection. Ten-day-old seedlings grown in 1/2 MS liquid medium were treated with mock or 2 × 10^8^ cfu/ml GV3101 suspension cells as the indicated time point. Then, total RNA was extracted, and the transcript level was analyzed by qRT-PCR for ACC synthetase *2* (*ACS2*) **(A)** and *ACS6* **(B)**. **(C, D)** The loss of the function of *MKK4* and *MKK5* compromises the expression of *ACS* genes during the agrobacteria inoculation. Total RNA was extracted from 10-day-old WT (Col-0) and *mkk4/5* double mutant seedlings treated as above for 3 h **(C)** or 24 h **(D)**, respectively. Relative gene expression levels are shown for: **(C)** *ACS2* and **(D)** *ACS6*. *UBQ10* was used as an internal control. **(E)** *Agrobacterium*-mediated transit transformation in an ET-defective mutant by using AGROBEST system. WT (Col-0), *acs2/6/4/5/9* mutant 10-day-old seedlings were inoculated with 10^8^ cfu/ml GV3101-pBISN1 suspension cells. GUS staining (left) and MUG assay (right) were measured at 3 dpi. **(F)** *Agrobacterium*-mediated stable transformation in ET defective mutant by using the tumorigenesis system. Root segments from the WT (Col-0) and *acs2/6/4/5/9* mutant were inoculated with 10^6^ cfu/ml of the *A. tumefaciens* strains A208. Tumors were scored at 30 days after infection. Values represent the average of three replicates with error bars indicating SD of the mean. “**” indicates a significant difference at *p* < 0.01. The scale bar indicates 5 mm.

As an important plant hormone, ET plays a major role in plant defense response to pathogen infection (Wang et al., 2002). MPK3/MPK6 regulate ET biosynthesis during pathogen attack (Han et al., 2010; Li et al., 2012). *Agrobacterium* has been found to regulate ET metabolism during the transformation process. Levels of the ET precursor ACC (1-amino-cyclopropane-1-carboxylate) were elevated during infection by agrobacteria (Lee et al., 2009). To clarify the roles of MKK4/MKK5 in regulatory pathways during the agrobacteria infection process, we investigated whether ET is involved in this process. Firstly, we measured the gene expression of rate-limiting enzyme of ET biosynthesis ACC synthetase (*ACS*) during agrobacteria infection. The results show that with agrobacteria inoculation, the ACC biosynthesis genes *ACS2, ACS4*, and *ACS6* were strongly induced compared with the mock treatment. These genes expression levels peaked at 6 hpi and decreased with further time after inoculation (Figures 9A,B; Supplementary Figure 6). This finding indicates that plants increased ET synthesis during agrobacteria infection.

We also detected the expression of *ACS* genes in *mkk4/5* double mutants. The results reveal that *ACS2* and *ACS4* gene expressions were impaired in *mkk4/5* double mutants inoculated with agrobacteria at 3 hpi while *ACS6* expression was impaired at 24 hpi (Figures 9C,D; Supplementary Figure 6). No difference in *ACS6* expression occurred in the WT (Col-0) mutant or *mkk4/5* double mutants at 3 hpi (Supplementary Figure 6), indicating that the regulation of *ACS6* gene expression occurs late in the agrobacteria infection process. These results imply that ET synthesis is regulated by MKK4/MKK5 at the *ACS* gene transcription level in the agrobacteria infection process.

To further explore the relationship between MKK4/MKK5 signaling and ET during *Agrobacterium-*mediated transformation, we applied exogenous ACC to the WT mutant and *mkk4/5* double mutants to determine whether ACC can rescue the transformation phenotype of *mkk4/5* double mutants. After treatment with ACC, *mkk4/5* double-mutant seedlings exhibited a two-fold decrease in the transformation frequency compared with the mock treatment (Supplementary Figure 6), indicating that the application of the exogenous ET precursor ACC can reverse the increased transformation phenotype of *mkk4/5*. Notably, ACC also caused the transformation frequency to decrease in the WT (Col-0) (Supplementary Figure 6), indicating that high ET levels had negative effects on the transformation by agrobacteria. To confirm the role of ET in transformation, we checked the transformation phenotype of ET synthesis defective mutant *acs2/6/4/5/9*. This mutant exhibited very low ET production compared with the WT (Col-0) (Li et al., 2012). Transient transformation frequency evaluation showed that *acs2/6/4/5/9* mutant seedlings had a greater GUS staining and 2.1-fold higher GUS activity than WT (Col-0) (Figure 9E). Tumorigenesis was analyzed as described earlier. WT and *acs2/6/4/5/9* mutant root segments were infected with the tumorigenic A. *tumefaciens* strain A208, and tumorigenesis efficiency was measured. The data show that the *acs2/6/4/5/9* mutant had more tumors in roots and exhibited a higher transformation frequency than WT (Figure 9F). These results suggest that ET synthesis is regulated by the MKK4/MKK5 signaling pathway and is involved in the transformation by *Agrobacterium*.

## Discussion

*Agrobacterium tumefaciens* has been widely used in plant transformation for decades, and the biological process of *Agrobacterium*-mediated transformation has been extensively studied (Gelvin, 2003; Citovsky et al., 2007). However, some aspects of this process, such as the mechanism of plant immunity to agrobacteria, and its roles in transformation remain unclear. In this study, we elucidated the *Arabidopsis* MKK4/5-MPK3/6 pathway, which regulates transformation by modulating plant immunity to *Agrobacterium*. As a plant pathogen, *Agrobacterium* triggers plant immunity characterized by MPK3/MKK6 activation, and defense-responsive gene induction (Figure 1). This process is similar to the responses to other plant pathogens such as *Pst* DC3000 (Nie and Xu, 2016).

Zipfel et al. (2006) demonstrated that plant immunity affects the process of transformation by *Agrobacterium*. Thus, plant transformation frequency can be increased in plants by manipulating plant immunity to *Agrobacterium*. The MKK4/5-MPK3/6 pathway is very important in the plant response defense to the pathogen *Pst* DC3000 (Nie and Xu, 2016). Here, we focused on MKK4/5-MPK3/6 pathway functions in *Agrobacterium*-triggered plant immunity and transformation. Our results reveal that the loss of the function of MKK4 and MKK5 compromised plant immunity, including MPK3/MPK6 activation, defense-responsive gene expression, ET synthesis, and SA accumulation. Deficient immunity accompanied a higher transformation frequency by *Agrobacterium* (Figure 3). Our results demonstrate that plant immunity to *Agrobacterium* affects transformation and that MKK4/MKK5 is essential for plant immunity signaling to *Agrobacterium*.

Ethylene and SA are the two plant hormones with very important roles in plant defense. Previous research has demonstrated that MPK3 and MPK6 regulate *Botrytis cinerea*-induced ET production by modulating ACC synthase at both the transcription and activity levels (Han et al., 2010; Li et al., 2012). Lee et al. (2009) reported that ET and SA accumulate before and after T-DNA integration during *Agrobacterium* transformation. In this study, we found that MKK4/MKK5 regulate agrobacteria-induced ET and SA biosynthesis (Figures 8, 9). Meanwhile, the exogenous application of ACC or SA can reverse the *mkk4/5* double-mutant phenotype (Supplementary Figures 5, 6). Notably, exogenous ACC or SA addition repressed the transformation in WT (Col-0) (Figures 8, 9). This result may indicate that exogenous ACC or SA strengthens the plant defense response or controls the virulence of *Agrobacterium*. ET production in plants was reported to suppress vir gene expression in agrobacteria during transformation (Nonaka et al., 2008). SA has an inhibitory effect on the virulence of agrobacteria by inhibiting the expression of the vir regulon on the Ti plasmid, which is essential for the transfer and integration of the T-DNA into the host genome (Yuan et al., 2007; Anand et al., 2008). These findings and our data reveal that the host plant is capable of controlling agrobacteria infection by modulating MKK4/MKK5-regulated ET and SA synthesis and defense signaling at an early stage.

Djamei et al. (2007) demonstrated that agrobacteria activate MPK3, which can phosphorylate VirE2 interacting protein 1 (VIP1, a bZIP transcription factor), resulting in the nuclear localization of VIP1 to support the transformation by *Agrobacterium*. In that study, the phosphorylation of VIP1 by MPK3 was found to be important for the transformation by *Agrobacterium*, so the authors suggested that agrobacteria hijack the MPK3-targeted VIP1 defense signaling pathway, allowing for the nuclear delivery of the T-DNA complex as a Trojan horse. Our data in this study show that MPK3 and MPK6 act downstream of MKK4/MKK5 to regulate the *Arabidopsis* defense response to agrobacteria. The loss of the function of *MPK3* and *MPK6* increased the transformation by *Agrobacterium* (Figure 5) while the *mpk3* single mutant showed no difference from the WT (Col-0) and the *mpk6* single mutant exhibited a slight increase in the transformation frequency (Supplementary Figure 2). Our results imply that MPK3 is not essential for transformation. Now, we have to mention the function of VIP1 in *Agrobacterium*-mediated transformation. As a transcription factor, VIP1 regulates plant stress gene expression and forms a ternary complex with VirE2 and importin α (Citovsky et al., 2004; Pitzschke et al., 2009). Tzfira et al. (2001, 2002) demonstrated that tobacco plants expressing an *Arabidopsis* VIP1 antisense construct were resistant to both transient and stable transformation by Agrobacterium, whereas the overexpression of VIP1 increased plant transformation by *Agrobacterium*. Based on those results, they concluded that VIP1 plays an important role in transformation. However, a recent study by Shi et al. (2014) using a quantitative *Arabidopsis* root assay indicated that the manipulation of VIP1 gene expression did not alter the susceptibility of *Arabidopsis* roots to *Agrobacterium*-mediated transformation. The authors suggested that VIP1 is not important for *Agrobacterium*-mediated transformation in *Arabidopsis*. Together, these results imply an unknown function of VIP1 in *Agrobacterium*-mediated transformation. A recent study by Wang et al. (2017) demonstrated that VirE2 interacts with multiple VIP1 homologs in the same host, indicating redundant functions of one or more proteins in the VIP1 subgroup. Our results regarding the MPK3 and MPK6 function in transformation show no difference between the WT and *mpk3* mutant (Supplementary Figure 3). We propose that this phenomenon can be readily explained. Firstly, MPK3 may not be the only upstream kinase (e.g., MPK4) that phosphorylates VIP1. Secondly, considering the redundant functions of VIP1 homologs, other proteins may be able to rescue the function of VIP1. Therefore, we interpret our results as not conflicting with those of previous studies.

This study elucidates the plant defense response process of signaling to *Agrobacterium*, which is mediated by the MKK4/5-MPK3/6 pathway and reveals the contribution of the plant defense response to the transformation frequency by *Agrobacterium*. Concerted research efforts on host plant defense signaling in response to *Agrobacterium* will build a strong foundation for modulating the *Agrobacterium*-mediated transformation process. Such a manipulation may support the development of more efficient transformation procedures for other crops.

## Data Availability Statement

The original contributions presented in the study are publicly available. This data can be found here: National Center for Biotechnology Information (NCBI) BioProject database under accession number GSE179628.

## Author Contributions

KD, TL, and LC designed the research. TL, LC, and JJ performed most of the research all authors contributed to material preparation and data analysis. KD wrote the paper. All authors read and approved the article.

## Funding

This research was supported by grants from the National Natural Science Foundation of China (31901957).

## Conflict of Interest

The authors declare that the research was conducted in the absence of any commercial or financial relationships that could be construed as a potential conflict of interest.

## Publisher's Note

All claims expressed in this article are solely those of the authors and do not necessarily represent those of their affiliated organizations, or those of the publisher, the editors and the reviewers. Any product that may be evaluated in this article, or claim that may be made by its manufacturer, is not guaranteed or endorsed by the publisher.

## Supplementary Material

Supplementary Figure 1 The activation of NtMEK2 in *Arabidopsis* increases the plant immunity and represses the *Agrobacterium*-mediated transformation. **(A)** NtMEK2-DD induction and MPK3/MPK6 activation in *NtMEK2-DD* transgenic seedlings. After pretreatment with dexamethasone (DEX), plant total protein was extracted from 10-day-old *NtMEK2-DD* transgenic seedlings grown in 1/2 Murashige and Skoog (MS) liquid medium. The induction of NtMEK2-DD expression and MPK3/MPK6 activation were detected by the immunoblot analysis using anti-FLAG and anti-pTEpY antibodies, respectively. Equal loading was confirmed by PONCEAU S staining. **(B)** The induction of expressions of defense genes in *NtMEK2-DD* transgenic seedlings. After pretreatment with DEX, total RNA was extracted from 10-day-old wild type (WT) (Col-0), *NtMEK2-DD* transgenic plants. Transcript levels were determined by quantitative real-time PCR (qRT-PCR) for *CRK11* (left) and *NHL10* (right). *UBQ10* was used as an internal control. **(C)** The evaluation of transformation frequency in *NtMEK2-DD* transgenic seedlings compared with WT (Col-0) using the AGROBEST system. Ten-day-old *Arabidopsis* WT (Col-0), *NtMEK2-DD* transgenic whole seedlings were infected with 10^8^ cfu/ml GV3101-pBISN1 suspension cells, and transformation frequency was measured by histochemical β-glucuronidase (GUS) staining (left) and 4-methylumbelliferyl-β-D-glucuronide (MUG) assay (right) at 3-day postinfection (dpi). Values represent the average of three replicates with error bars indicating SD of the mean. “^**^” indicates a significant difference at *p* < 0.01. The scale bar indicates 5 mm.

Supplementary Figure 2 *Agrobacterium*-mediated transformation in the *mpk3* and *mpk6* single mutant. *Agrobacterium*-mediated transit transformation by using the AGROBEST system. WT (Col-0), 10-day-old seedlings of *mpk3-1* and *mpk6-2* mutants were inoculated with 10^8^ cfu/ml GV3101-pBISN1 suspension cells. GUS staining (left) and MUG assay (right) were measured at 3 dpi. Values represent the average of three replicates with error bars indicating SD of the mean. The scale bar indicates 5 mm.

Supplementary Figure 3 Global transcriptome indicates that agrobacteria trigger plant defense pathways. Gene ontology (GO) biological process categories in WT induced by agrobacteria treatment. Sets of genes used to determine significantly enriched GO terms were those found to be differentially expressed at *p* < 0.05 significance level and showed at least a two-fold change in expression. The included categories are those found to be significantly enriched (*p* < 0.01) among the upregulated gene in agrobacteria treatment.

Supplementary Figure 4 Agrobacteria*-*triggered plant defense pathways are dependent on MKK4/MKK5. GO biological process categories are induced by agrobacteria in WT while no changes in *mkk4/5* double-mutant plants.

Supplementary Figure 5 Salicylic acid (SA) reverses the *mkk4/5* phenotype of transformation. **(A)** SA synthesis gene expression in WT after agrobacteria infection. Ten-day-old seedlings grown in 1/2 MS liquid medium were treated with mock or 2 × 10^8^ cfu/ml GV3101 suspension cells as the indicated time point. Then, total RNA was extracted, and the transcript level was analyzed by qRT-PCR for *AtICS1* (left) and *AtEDS5* (right). **(B)** Exogenous application of SA reversed the increased transformation phenotype of the *mkk4/5* double mutant. Ten-day-old WT (Col-0) and *mkk4/5* double-mutant seedlings grown in 1/2 liquid MS medium were pretreated with SA (10 μM) for overnight and then were infected with 10^8^ cfu/ml GV3101-pBISN1 cells. GUS staining (left) and MUG assay (right) were measured at 3-day post-infiltration. Values represent the average of three replicates with error bars indicating SD of the mean. “^**^” indicates a significant difference at *p* < 0.01. The scale bar indicates 5 mm.

Supplementary Figure 6 1-Amino-cyclopropane-1-carboxylic acid (ACC) reverses the *mkk4/5* phenotype of transformation. **(A)** *ACS4* gene expression in WT and *mkk4/5* double mutants during agrobacteria infection. Ten-day-old seedlings grown in 1/2 MS liquid medium were treated with mock or 2 × 10^8^ cfu/ml GV3101 suspension cells as the indicated time point. Then, total RNA was extracted, and the transcript level was analyzed by qRT-PCR. **(B)** *ACS6* gene expression in WT and *mkk4/5* double mutants after 3 h post-agrobacteria infection. **(C)** Exogenous application of ACC reversed the increased transformation of *mkk4/5* double mutants. Ten-day-old WT (Col-0) and *mkk4/5* double-mutant seedlings grown in 1/2 liquid MS medium were pretreated with ACC (10 μM) for overnight and then were infected with 10^8^ cfu/ml GV3101-pBISN1 cells. GUS staining (left) and MUG assay (right) were measured at 3-day post-infiltration. Values represent the average of three replicates with error bars indicating SD of the mean. “^**^” indicates a significant difference at *p* < 0.01. The scale bar indicates 5 mm.

Supplementary Materials 1 and 2 Agrobacteria-responsive genes (ARGs) in Col-0 and *mkk4/5* mutant from RNA-seq data for GO enrichment analysis.

Supplementary Table 1 Primers used in this study.
