# Supplementary figures and images for: MKK4/5-MPK3/6 Cascade Regulates Agrobacterium-Mediated Transformation by Modulating Plant Immunity in Arabidopsis
Source: Front Plant Sci. 2021 Sep 30;12:731690. doi: 10.3389/fpls.2021.731690 (PMC8514879; doi:10.3389/fpls.2021.731690)

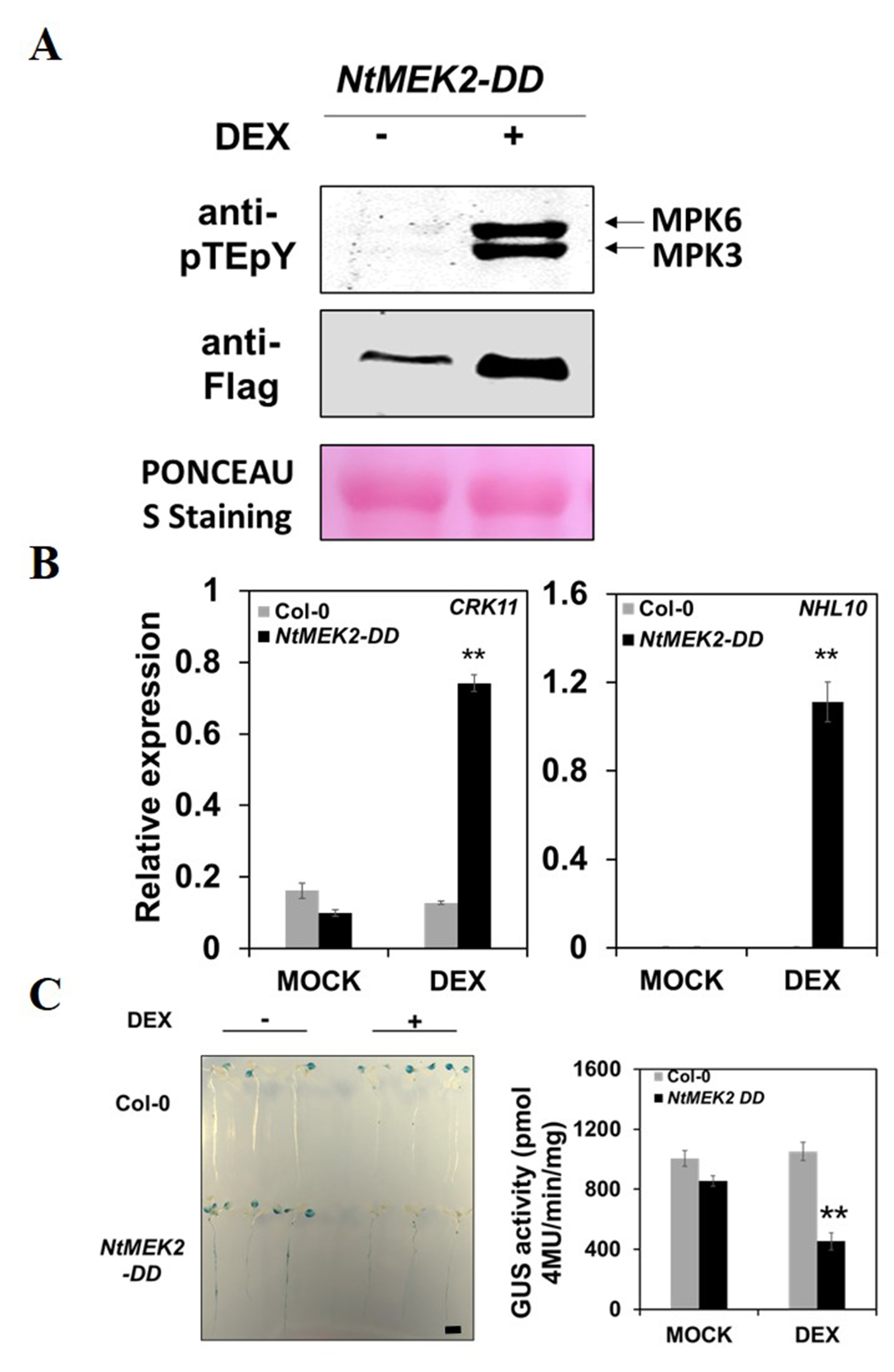

Supplement: Supplementary Figure 1 — The activation of NtMEK2 in Arabidopsis increases the plant immunity and represses the Agrobacterium-mediated transformation. (A) NtMEK2-DD induction and MPK3/MPK6 activation in NtMEK2-DD transgenic seedlings. After pretreatment with dexamethasone (DEX), plant total protein was extracted from 10-day-old NtMEK2-DD transgenic seedlings grown in 1/2 Murashige and Skoog (MS) liquid medium. The induction of NtMEK2-DD expression and MPK3/MPK6 activation were detected by the immunoblot analysis using anti-FLAG and anti-pTEpY antibodies, respectively. Equal loading was confirmed by PONCEAU S staining. (B) The induction of expressions of defense genes in NtMEK2-DD transgenic seedlings. After pretreatment with DEX, total RNA was extracted from 10-day-old wild type (WT) (Col-0), NtMEK2-DD transgenic plants. Transcript levels were determined by quantitative real-time PCR (qRT-PCR) for CRK11 (left) and NHL10 (right). UBQ10 was used as an internal control. (C) The evaluation of transformation frequency in NtMEK2-DD transgenic seedlings compared with WT (Col-0) using the AGROBEST system. Ten-day-old Arabidopsis WT (Col-0), NtMEK2-DD transgenic whole seedlings were infected with 108 cfu/ml GV3101-pBISN1 suspension cells, and transformation frequency was measured by histochemical β-glucuronidase (GUS) staining (left) and 4-methylumbelliferyl-β-D-glucuronide (MUG) assay (right) at 3-day postinfection (dpi). Values represent the average of three replicates with error bars indicating SD of the mean. “**” indicates a significant difference at p < 0.01. The scale bar indicates 5 mm. [file Image_1.JPEG]

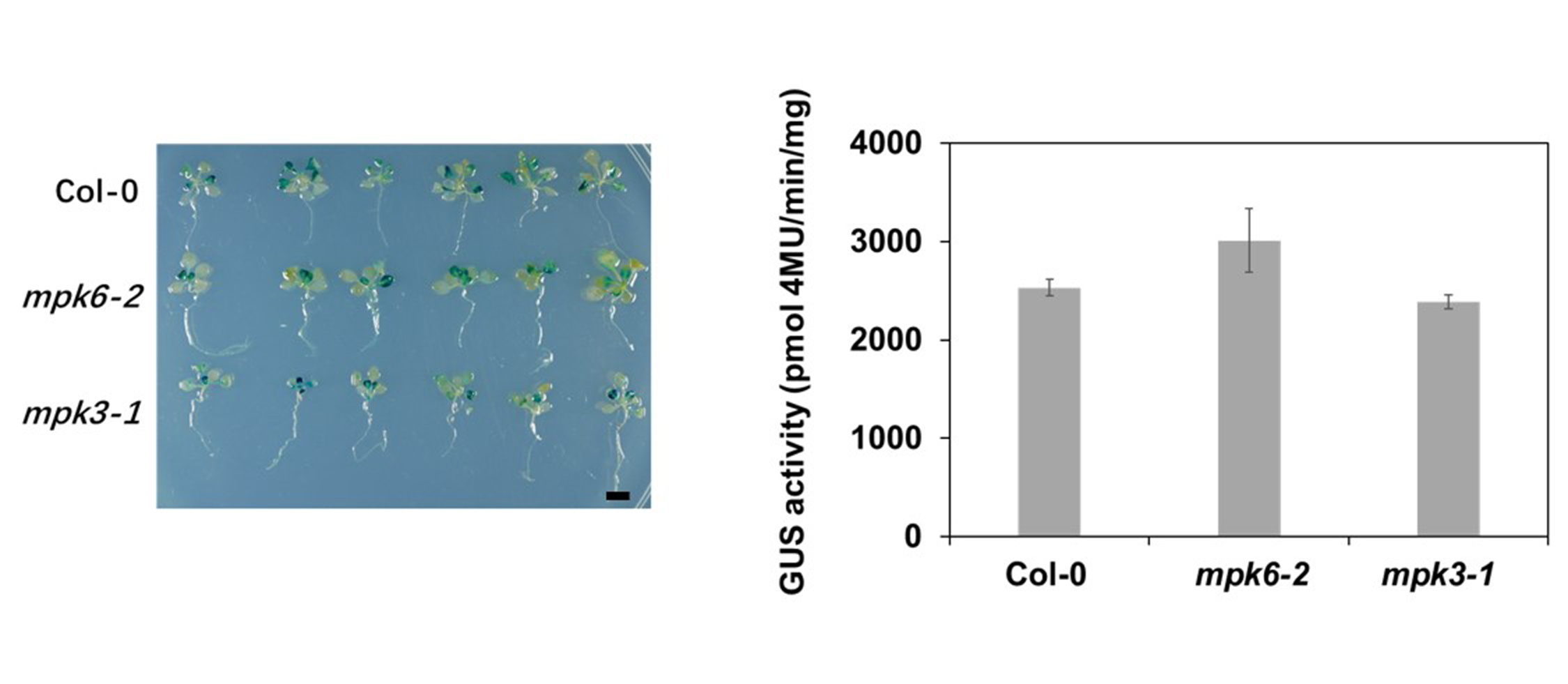

Supplement: Supplementary Figure 2 — Agrobacterium-mediated transformation in the mpk3 and mpk6 single mutant. Agrobacterium-mediated transit transformation by using the AGROBEST system. WT (Col-0), 10-day-old seedlings of mpk3-1 and mpk6-2 mutants were inoculated with 108 cfu/ml GV3101-pBISN1 suspension cells. GUS staining (left) and MUG assay (right) were measured at 3 dpi. Values represent the average of three replicates with error bars indicating SD of the mean. The scale bar indicates 5 mm. [file Image_2.JPEG]

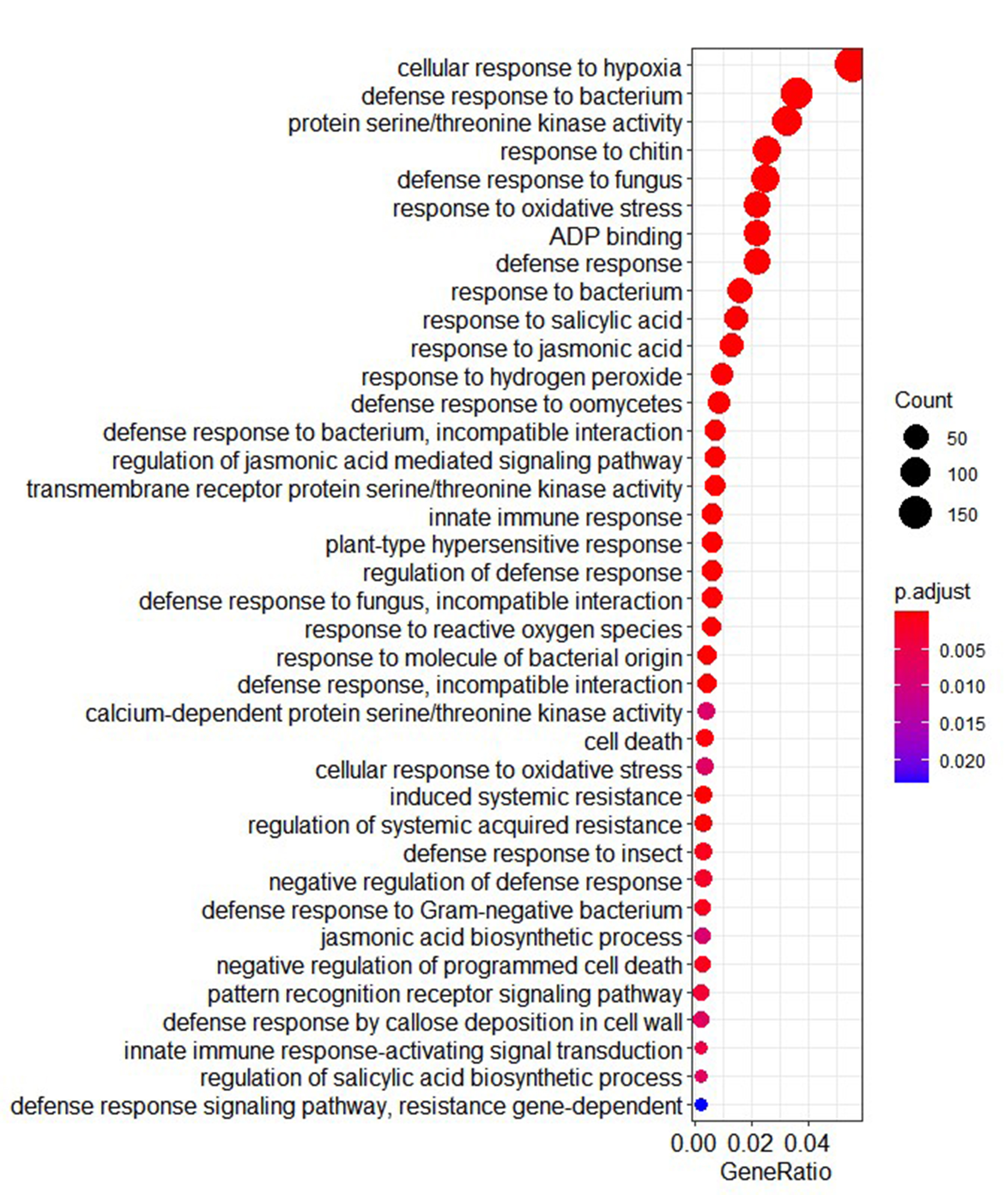

Supplement: Supplementary Figure 3 — Global transcriptome indicates that agrobacteria trigger plant defense pathways. Gene ontology (GO) biological process categories in WT induced by agrobacteria treatment. Sets of genes used to determine significantly enriched GO terms were those found to be differentially expressed at p < 0.05 significance level and showed at least a two-fold change in expression. The included categories are those found to be significantly enriched (p < 0.01) among the upregulated gene in agrobacteria treatment. [file Image_3.JPEG]

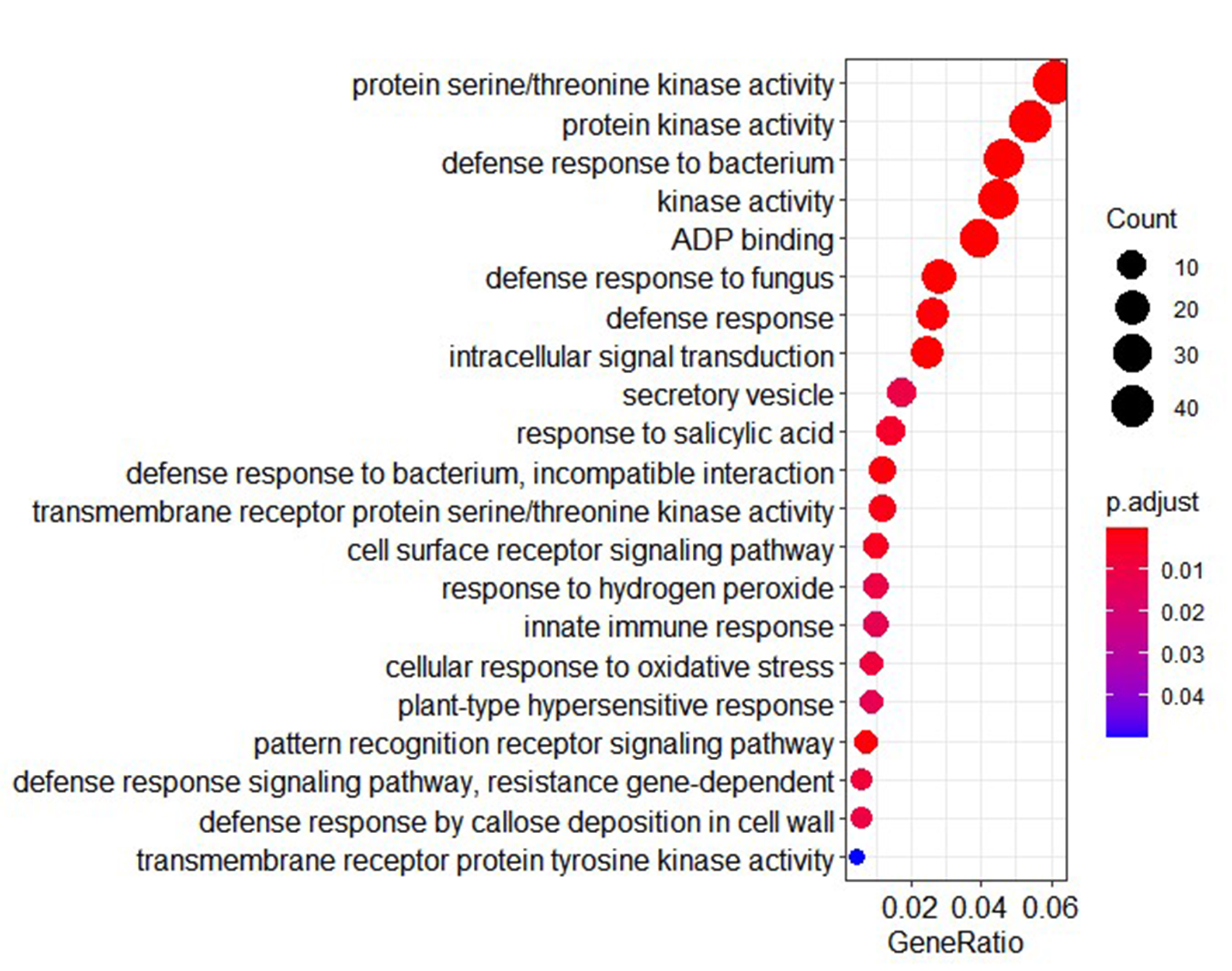

Supplement: Supplementary Figure 4 — Agrobacteria-triggered plant defense pathways are dependent on MKK4/MKK5. GO biological process categories are induced by agrobacteria in WT while no changes in mkk4/5 double-mutant plants. [file Image_4.JPEG]

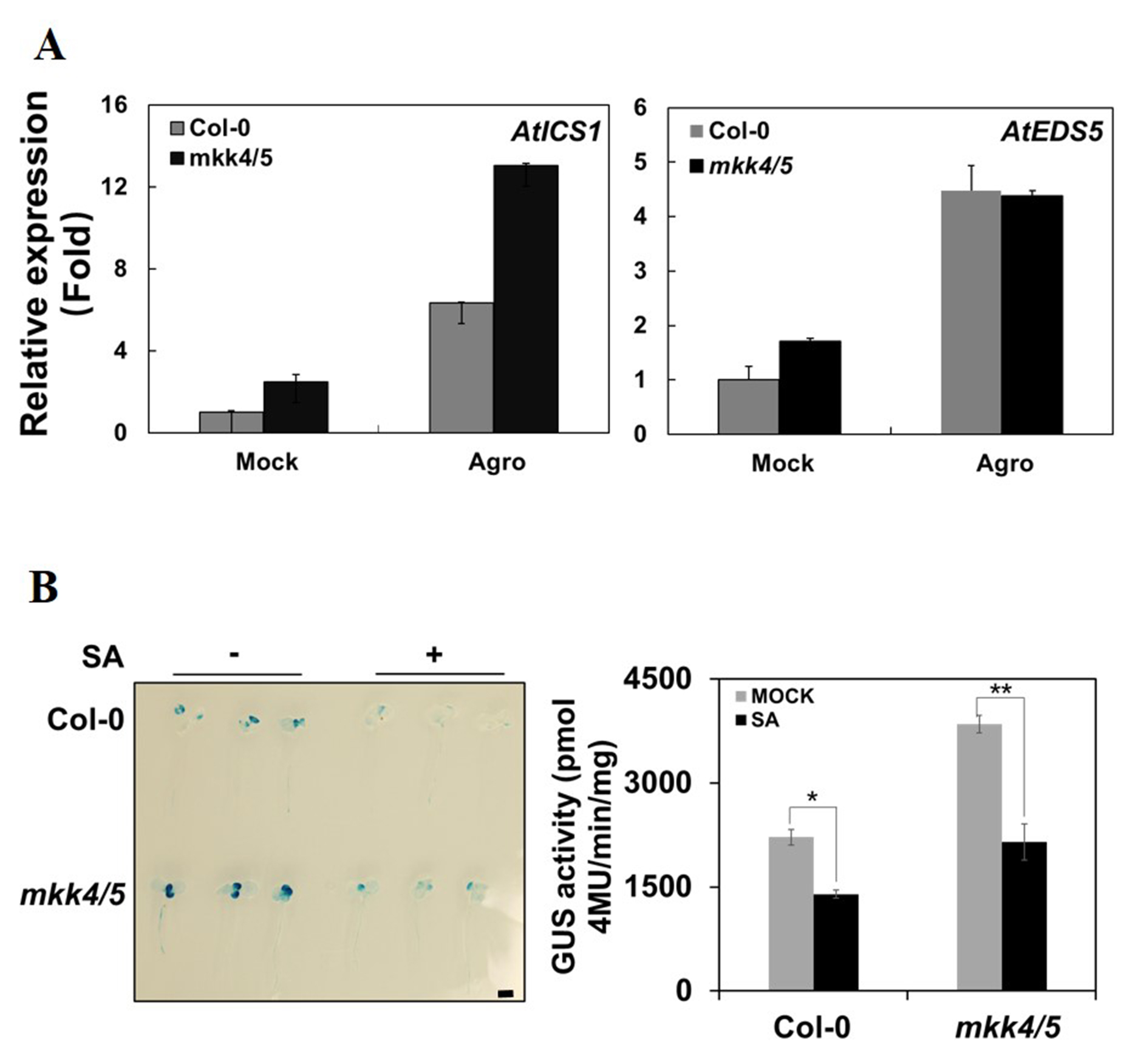

Supplement: Supplementary Figure 5 — Salicylic acid (SA) reverses the mkk4/5 phenotype of transformation. (A) SA synthesis gene expression in WT after agrobacteria infection. Ten-day-old seedlings grown in 1/2 MS liquid medium were treated with mock or 2 × 10^8^ cfu/ml GV3101 suspension cells as the indicated time point. Then, total RNA was extracted, and the transcript level was analyzed by qRT-PCR for AtICS1 (left) and AtEDS5 (right). (B) Exogenous application of SA reversed the increased transformation phenotype of the mkk4/5 double mutant. Ten-day-old WT (Col-0) and mkk4/5 double-mutant seedlings grown in 1/2 liquid MS medium were pretreated with SA (10 μM) for overnight and then were infected with 108 cfu/ml GV3101-pBISN1 cells. GUS staining (left) and MUG assay (right) were measured at 3-day post-infiltration. Values represent the average of three replicates with error bars indicating SD of the mean. “**” indicates a significant difference at p < 0.01. The scale bar indicates 5 mm. [file Image_5.JPEG]

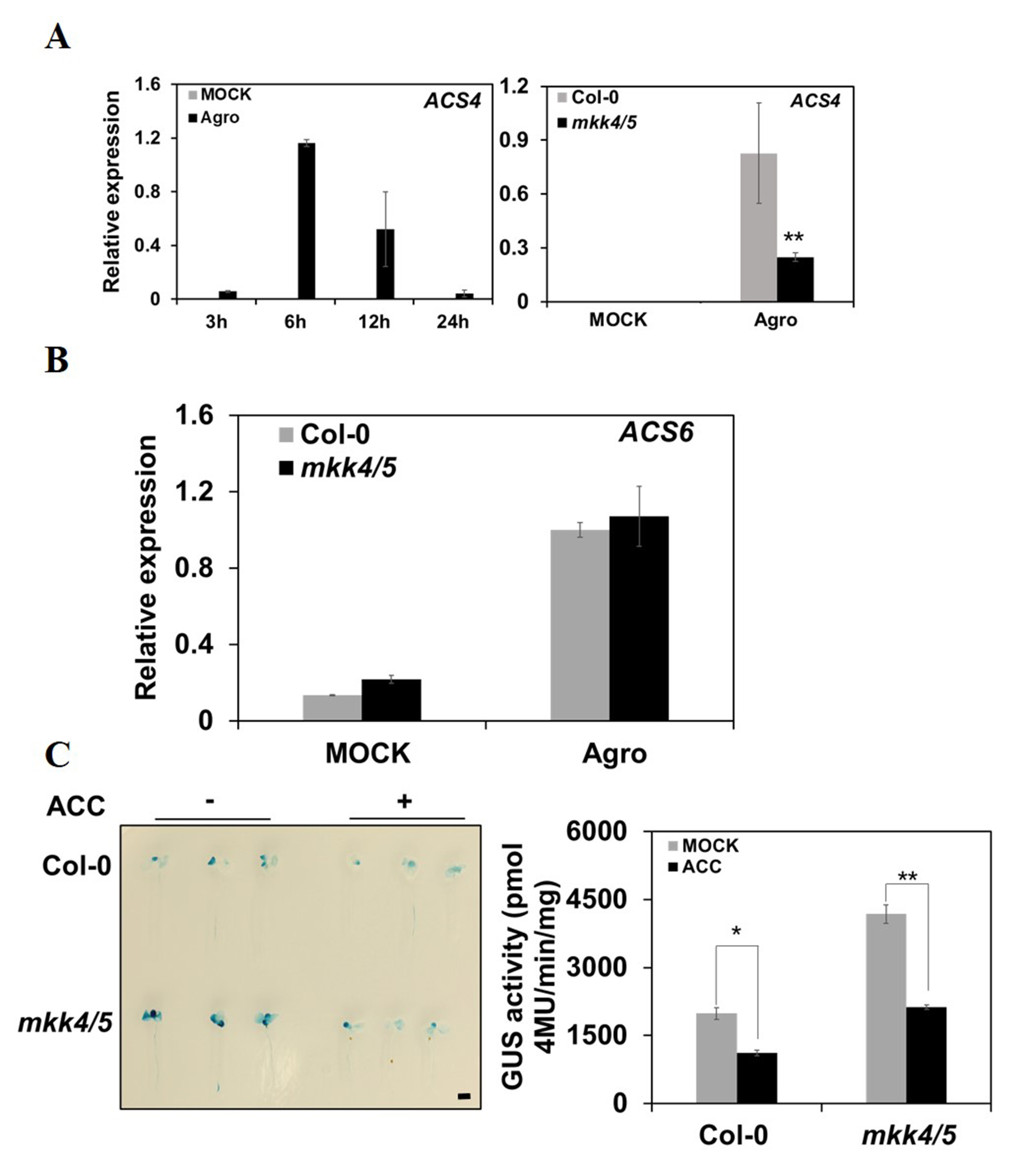

Supplement: Supplementary Figure 6 — 1-Amino-cyclopropane-1-carboxylic acid (ACC) reverses the mkk4/5 phenotype of transformation. (A) ACS4 gene expression in WT and mkk4/5 double mutants during agrobacteria infection. Ten-day-old seedlings grown in 1/2 MS liquid medium were treated with mock or 2 × 10^8^ cfu/ml GV3101 suspension cells as the indicated time point. Then, total RNA was extracted, and the transcript level was analyzed by qRT-PCR. (B) ACS6 gene expression in WT and mkk4/5 double mutants after 3 h post-agrobacteria infection. (C) Exogenous application of ACC reversed the increased transformation of mkk4/5 double mutants. Ten-day-old WT (Col-0) and mkk4/5 double-mutant seedlings grown in 1/2 liquid MS medium were pretreated with ACC (10 μM) for overnight and then were infected with 108 cfu/ml GV3101-pBISN1 cells. GUS staining (left) and MUG assay (right) were measured at 3-day post-infiltration. Values represent the average of three replicates with error bars indicating SD of the mean. “**” indicates a significant difference at p < 0.01. The scale bar indicates 5 mm. [file Image_6.JPEG]
