# Supplementary material for: MKK4/5-MPK3/6 Cascade Regulates Agrobacterium-Mediated Transformation by Modulating Plant Immunity in Arabidopsis
Source: Front Plant Sci. 2021 Sep 30;12:731690. doi: 10.3389/fpls.2021.731690 (PMC8514879; doi:10.3389/fpls.2021.731690)
Supplement: Supplementary Table 1 — Primers used in this study. [file Table_1.docx]

| Gene name or ID | Forward | Reverse |
| --- | --- | --- |
| *FRK1* | ATCTTCGCTTGGAGCTTCTC | TGCAGCGCAAGGACTAGAG |
| *NHL10* | ATGGCTGCTGAACAACCTCT | CGGTCACGTGAAACTTGATG |
| *At2g17740* | TGCTCCATCTCTCTTTGTGC | ATGCGTTGCTGAAGAAGAGG |
| *CRK11* | ACTAGTACTG CAGGAAACTT | CTGGTAGAGT AATTGTGTTA |
| *ACS2* | GGATGGTTTAGGATTTGCTTTG | GCACTCTTGTTCTGGATTACCTG |
| *ACS4* | AACAACCTTGTGCTCACTGCT | AGATCCCTATCAAACCCTGGA |
| *ACS6* | GTTCCAACCCCTTATTATCC | CCGTAATCTTGAACCCATTA |
| *SID2* | GCTTGGCTAGCACAGTTACAGC | CACTGCAGACACCTAATTGAGTCC |
| *EDS1*  *UBQ10* | TGTGGAAATGGCTGTGAGGA  CCTTGTATAATCCCTGATGA | TTCTCTCCCAAGTGCGTGAC  AACAGGAACGGAAACATAGT |
| *AtMKK4* | CATATGAGACCGATTCAATCGCC | ACTAGTAAAATTCAGAGACCCTCC |
| *AtMKK5* | CATATGAAACCGATTCAATCTCCTTC | ATCCAGCTAAAATCACTCTTAAACCA |
| *NtMEK2* | ATGCGACCTCTTCAACCACC | TTAAGAAGAAAAATGAGGAGGT |

**Table S1 Primers used for qRT-PCR and construction in this study**
